# Annexin A2 and lamin B join membrane recycling compartments for the assembly of biomolecular condensates operating in mitotic partitioning

**DOI:** 10.3389/fcell.2025.1744307

**Published:** 2026-01-15

**Authors:** Ann Kari Grindheim, Hege Dale, Josef Novák, Sudarshan Shantinath Patil, Anni Vedeler, Jaakko Saraste

**Affiliations:** 1 Department of Biomedicine, Bergen, Norway; 2 Molecular Imaging Center (MIC), University of Bergen, Bergen, Norway

**Keywords:** Annexin A2, biomolecular condensates, ER-Golgi intermediate compartment (IC or ERGIC), lamin B, mitosis, RAB1, Rab11, recycling endosome (RE)

## Abstract

Localization of the actin-, lipid- and mRNA-binding protein Annexin A2 (AnxA2) in dividing cells revealed its presence in large spherical structures which are confined to the cell periphery and frequently co-align with astral microtubules. These structures appear during prometaphase and disappear at telophase, coinciding with the mitotic breakdown and subsequent reformation of the nuclear lamina and envelope. Their size increases as cells progress to anaphase, while their number decreases, suggesting that they are capable of fusion. Treatment of cells with the aliphatic alcohol propylene glycol led to rapid and reversible disassembly of the structures, providing further evidence that they correspond to biomolecular condensates. Notably, the condensates enclose compartments involved in biosynthetic or endocytic membrane recycling – defined by Rab1, Rab11, or endocytosed transferrin–but lack other membrane organelles, indicating that they may serve as mitotic reservoirs for selected endomembranes. Additionally, the condensates incorporate lamin B, which connects with the pericentrosomal membrane recycling compartments during prometaphase, when the nuclear lamina disassembles in conjunction with centrosome separation. These findings show similarities between the peripheral mitotic condensates and the membranous lamin B spindle matrix which has been proposed to act in spindle organization and organelle inheritance. The separating daughter cells at late anaphase contain equal numbers of the condensates, in accordance with their potential role in mitotic partitioning of endomembranes and other cytoplasmic components.

## 1 Introduction

Cell division involves an extensive reorganization of the cell’s internal architecture and a profound alteration in cell shape (Champion et al., 2017; Carlton et al., 2020). With respect to the cytoskeleton, as cells enter mitosis, the typically radial array of microtubules (MTs) characteristic of interphase cells is reorganized into the mitotic spindle–a bipolar structure that provides the framework for chromosome alignment and segregation. Astral MTs, which connect the spindle poles to the cell cortex, along with the MT-dependent motor protein dynein, play a crucial role in determining the correct positioning of the spindle, thereby influencing the fidelity of chromosome segregation (Di Pietro et al., 2016). Furthermore, the cortical network of actin filaments and associated proteins undergoes significant remodelling during mitosis, creating specialized attachment sites for the astral MTs and facilitating the rounding of the cells (Théry and Bornens, 2008; Champion et al., 2017).

In addition to chromosomes, cytoplasmic components – including the various organelles of the endomembrane system – must be evenly distributed between the daughter cells. While the segregation of genetic material is well known, the mechanisms of organelle inheritance remain controversial (Carlton et al., 2020). Moreover, the coordination between these two processes is poorly understood. For proper partitioning, the single-copy organelles of the secretory pathway, the endoplasmic reticulum (ER) and Golgi apparatus, must undergo remodelling or complete disassembly (Champion et al., 2017; Ayala et al., 2020). The mitotic ER network is typically excluded from the spindle region (Ellenberg et al., 1997; Schlaitz et al., 2013) and adopts a predominantly tubular or sheet-like organization, depending on the cell type (Puhka et al., 2007; Lu et al., 2009; Puhka et al., 2012; Kumar et al., 2021). The nuclear envelope (NE) – a subdomain of the ER–breaks down during prometaphase, coinciding with the disassembly of the nuclear lamina. Solubilized A-type lamins are dispersed throughout the cytoplasm, while lipid-linked B-type lamins remain membrane-bound. Like integral NE components, B-type lamins are thought to redistribute to the mitotic ER (Gerace et al., 1978; Mall et al., 2012; Ungricht and Kutay, 2017). Alternatively, based on studies of *Drosophila* and *Xenopus* egg extracts, the release of lamin B, along with other nuclear proteins, has been associated with the formation of a membranous matrix that regulates the assembly and function of the mitotic spindle (Tsai et al., 2006; Ma et al., 2009; Zheng, 2010; Johansen et al., 2011; Schweizer et al., 2014; Scholey, 2025). Although the exact nature of this lamin B spindle matrix remains enigmatic, its formation is believed to initiate upon NE breakdown and involve dynein-dependent transport of lamin B towards the separating spindle poles (Beaudouin et al., 2002; Salina et al., 2002).

As cells enter mitosis, the Golgi ribbon initially undergoes fragmentation, followed by the vesiculation of the separated cisternal stacks (Ayala et al., 2020). The disassembled Golgi elements may retain their autonomy and serve as templates for organelle reassembly during mitotic exit (Seemann et al., 2002). Alternatively, Golgi enzymes could be recycled back to the ER and, similar to integral NE proteins, partition as ER components due to a mitotic block in ER exit (Zaal et al., 1999). In contrast to the Golgi, the intermediate compartment (IC) involved in ER-Golgi trafficking retains many of its compositional and structural properties, as well as its association with spindle MTs (Marie et al., 2012). Additionally, the IC maintains its connection with recycling endosomes (REs) at the spindle poles (Marie et al., 2009; Marie et al., 2012; Takatsu et al., 2013; Hehnly and Doxsey, 2014), allowing for the coordinated partitioning of these pericentrosomal compartments at the onset of mitosis, in a process which is linked to centrosome separation and spindle formation (Marie et al., 2012; Takatsu et al., 2013; Saraste and Prydz, 2019).

The correct orientation of the spindle apparatus relies on a conserved protein complex that mediates the cortical anchoring of astral MTs (di Pietro et al., 2016). One key component of this complex is AnxA2 (Pascal et al., 2022), a multifunctional protein that interacts with actin and negatively charged phospholipids–such as phosphatidylinositol 4,5-bisphosphate [PI(4,5)P_2_] – in a Ca^2+^-dependent manner (Hayes et al., 2004a; Rescher et al., 2004; Gerke et al., 2005; Grieve et al., 2012; Bharadwaj et al., 2013). Given these properties and its accumulation beneath the plasma membrane (PM) in interphase cells, AnxA2 has been implicated in cortical actin remodelling (Hayes et al., 2004b; Grieve et al., 2012). In addition, AnxA2 plays a role in mRNA localization and translation, as well as secretory and endocytic membrane trafficking (Mickleburgh et al., 2005; Grieve et al., 2012; Vedeler et al., 2012; Grindheim et al., 2016; Strand et al., 2021; Grindheim et al., 2023; Gerke et al., 2024). In the endocytic pathway, AnxA2 has been shown to associate with REs and multivesicular bodies (MVBs) (Zeuschner et al., 2001; Mayran et al., 2003; Zobiack et al., 2003; Delevoye et al., 2016). It is also further targeted to the luminal exosomes of the MVBs (Valapala and Vishwanatha, 2011; Grindheim et al., 2016; Grindheim and Vedeler, 2016).

The function of AnxA2 is essential for successful cell division. It localises to the intercellular bridge that connects the forming daughter cells (Skop et al., 2004) and is necessary during the early stages of cytokinesis (Benaud et al., 2015). This requirement may be linked to its interactions with PI(4,5)P_2_-enriched membrane domains and/or its association with Rab11-positive REs which are involved in membrane delivery to the intercellular bridge (Fielding et al., 2005; Wilson et al., 2005; Benaud and Prigent, 2016). In the present study, we employed special fixation conditions to investigate the localization of AnxA2 during the early phases of cell division, revealing its association with large spherical structures–up to 2 μm in diameter–that transiently appear at the periphery of mitotic cells between prometaphase and telophase. We provide evidence that these structures represent biomolecular condensates–organelles with liquid-like properties that form through phase separation and play crucial roles in subcellular organization (Banani et al., 2017). The association of biosynthetic and endocytic membrane recycling compartments–namely the IC and REs–with these peripheral condensates suggests their involvement in the mitotic storage and segregation of selected endomembranes, as well as in the regulation of cell surface area (Boucrot and Kirchhausen, 2007). Furthermore, based on their content of lamin B, the condensates are likely to correspond to the membrane-containing spindle matrix which has been proposed to function in spindle regulation and coordination of mitotic partitioning events (Zheng, 2010; Johansen et al., 2011; Schweizer et al., 2014).

## 2 Results

### 2.1 Localisation of AnxA2 in mitotic cells

**FIGURE 1:**
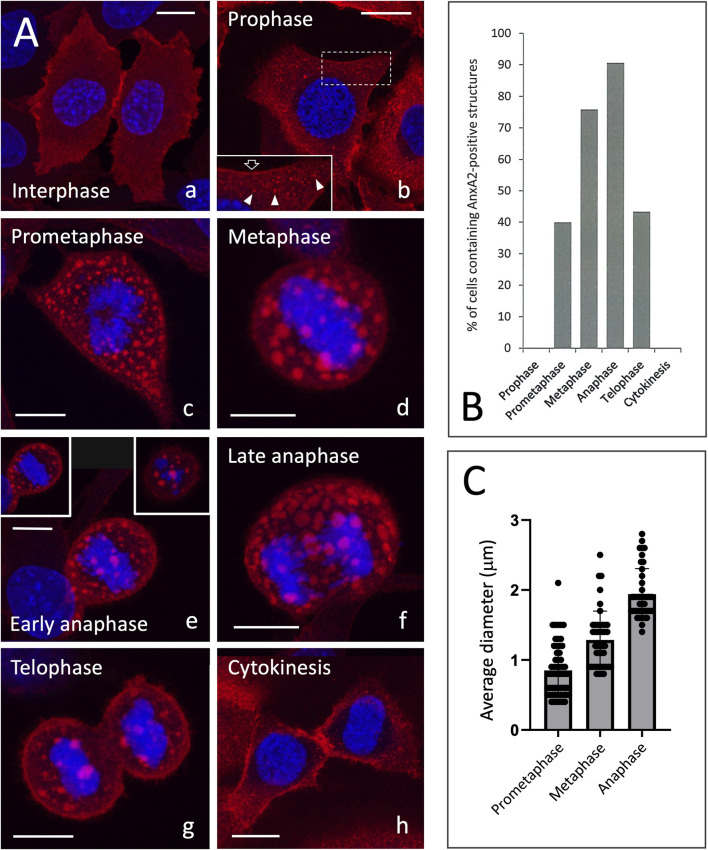
**(A)** *Localisation of AnxA2 during the cell cycle*. NRK cells were fixed with PLP and permeabilised with saponin. Following exposure of antigenic sites by treatment with guanidine-HCl, the cells were immunolabelled using monoclonal antibodies against AnxA2 (red). Cells at interphase (a), the different phases of mitosis (b–g), or cytokinesis (h) were identified by DAPI staining of DNA (blue). Small AnxA2-positive puncta appear during prophase (b, inset; white arrowheads) and the cortical signal appears to be diminished (open arrow). The images are maximum intensity projections, except the insets in panel e, which represent single optical sections from the middle (left inset) or top (right inset) of an early anaphase cell, showing the characteristic peripheral localization of the large AnxA2-positive structures. Scale bars: 10 µm. **(B)** *Transient appearance of the AnxA2-positive structures during mitosis*. The cells were processed and labelled using monoclonal AnxA2 antibodies and DAPI as described above. The percentages of cells at different phases of mitosis or cytokinesis, containing the large AnxA2-positive structures, were determined based on the examination of a large number of cells (n = 600). Note that the great majority of cells at meta- and anaphase are positive for these structures, indicating their structural preservation. **(C)** *The size of the mitotic structures increases during mitosis.* The results are based on the measurement of the diameters of 57 (prometaphase), 44 (metaphase) and 41 (anaphase) mitotic structures. The average diameters, standard deviations and size distribution of the structures in the three mitotic stages are indicated.

To investigate the detailed localisation of AnxA2 during cell division we used unsynchronized cultures of normal rat kidney (NRK) cells, which exhibit a high mitotic index and are commonly used in studies of mitosis (Beaudouin et al., 2002; Salina et al., 2002; Seemann et al., 2002; Marie et al., 2012). Using a standard immunofluorescence protocol involving para-formaldehyde (PFA) fixation and saponin-permeabilization, we observed cortical accumulation of AnxA2 in the mitotic cells, similar to the distribution seen at interphase (Grieve et al., 2012; Grindheim et al., 2016). Interestingly, AnxA2 was also detected in distinct peripheral structures; however, their size and appearance varied significantly between different experiments, indicating poor preservation (data not shown). To enhance the visualization of these structures, we switched to paraformaldehyde-lysine-periodate (PLP) fixation (McLean and Nakane, 1974) which provides cross-linking and improves structural preservation (Brown and Farquhar, 1989). Following PLP fixation, the AnxA2-positive spherical structures were consistently larger and displayed a smooth appearance, particularly when fixation was performed on ice (Figure 1A; see Materials and methods). Confirming their authenticity, similar structures were observed in other cultured cell types, including baby hamster kidney (BHK21) cells, human keratinocytes (HaCaT), human retinal epithelial cells (RPE-1) and rat embryonal fibroblasts (REF52) (Supplementary Figure S1).

By contrast, similar AnxA2-containing mitotic structures were not detected in transformed human HeLa cells, aligning with previous findings (Pascal et al., 2022). However, as discussed in more detail below, the presence of transferrin (Tfn)-positive peripheral puncta may indicate that these structures also exist in HeLa cells (Supplementary Figure S1, panel E; white arrowheads) but may be poorly preserved under the fixation and permeabilization conditions used. Indeed, previous electron microscopy studies involving glutaraldehyde fixation reported the presence of distinct membrane clusters at the periphery of mitotic HeLa cells (Tooze and Hollinshead, 1992). In addition to cortical localization and large size, their labelling by endocytosed Tfn and resistance to BFA (see below) suggest that these tubular clusters correspond to the AnxA2-positive mitotic structures described in the present study.

Examination of the PLP-fixed NRK cells at various phases of the cell cycle confirmed that the novel AnxA2-containing structures are specific to mitotic cells (Figure 1A). To gain a better understanding of their nature, we quantified their presence in a large number of dividing cells, using DNA staining (DAPI) to identify cells at different phases of mitosis or cytokinesis. As shown in Figure 1B, the large AnxA2-positive structures are still absent in prophase cells which, however, contain small AnxA2-positive puncta (Figure 1A, panel b; inset) – but readily detectable during prometaphase, with approximately 40% of cells being positive. The percentage of positive cells then increases from metaphase (about 75%) to anaphase (about 90%), before decreasing again at telophase (around 40%). Cells undergoing cytokinesis did not show these structures, as illustrated in Figure 1A (panel h). In summary, these AnxA2-positive structures represent transient mitosis-specific assemblies that are detectable from prometaphase to telophase.

As mentioned above, the intracellular distribution of AnxA2 already begins to change during prophase with the appearance of small cytoplasmic puncta (Figure 1A; panels b). At prometaphase, however, large AnxA2-containing structures – exhibiting predominantly peripheral localization and variable size–emerge simultaneously with cell rounding (Figure 1A; panel c). Z-stacks generated from cells at later stages of mitosis demonstrated that these structures are regularly spaced and evenly distributed throughout the cell periphery, primarily lining the cytoplasmic aspect of the PM (Figure 1A; panel e, insets). The predominantly peripheral localization of the structures, as well as their absence from the spindle region is also illustrated in Supplementary Movie-1. Interestingly, their average size increases from 0.8 µm to 1.9 µm in diameter as the cells progress from prometaphase to anaphase (Figure 1C). The observed gradual increase in size appears to favour fusion over ongoing disassembly and reformation of the structures (Figure 1C). Furthermore, the concurrent decrease in their number (Figure 1A, compare, e.g. panels c and d – for quantification, see Figure 8A) suggests that these structures are capable of fusion.

### 2.2 The AnxA2-positive structures contain specific membranes

**FIGURE 2:**
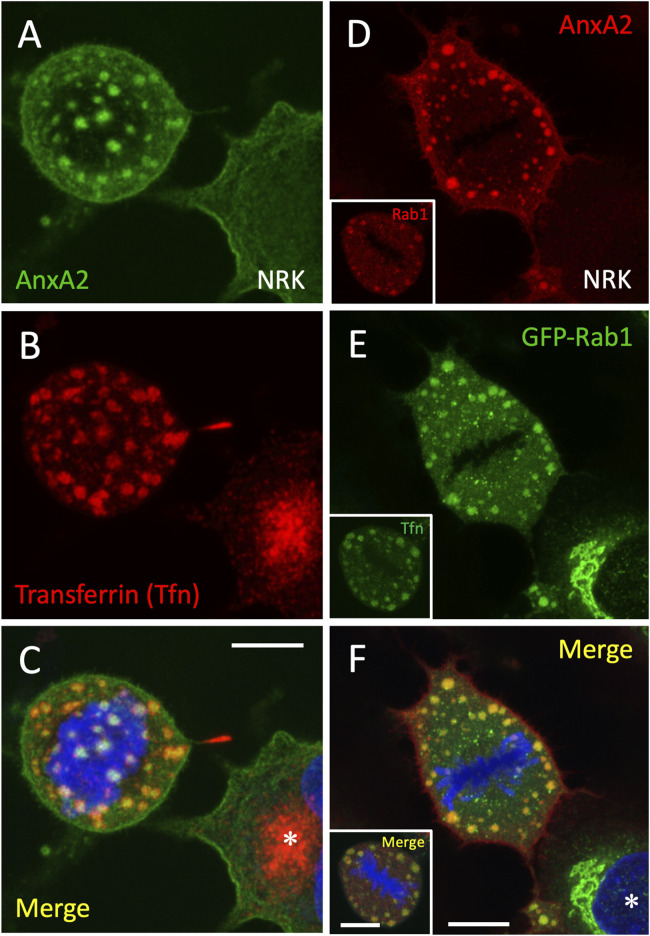
*Association of endocytic and biosynthetic membrane recycling compartments with the AnxA2-positive mitotic structures*. **(A–C)** NRK cells were subjected to uptake of Alexa-Fluor 594-transferrin (Tfn; red) to label the recycling endosomal system, followed by fixation with PLP and staining with antibodies against AnxA2 (green) and DAPI (blue) as described for Figure 1. **(D–F)** NRK cells expressing the IC marker GFP-Rab1 (green) were stained for AnxA2 (red). The insets show NRK cells subjected to Alexa-Fluor 488 Tfn-uptake (green) and stained after fixation for endogenous Rab1 (red). **(A–C)** show the top of the cell at early anaphase while the images in **(D–F)** represent maximum intensity projections from the middle of the metaphase cell. Interphase cells are marked with asterisks. Scale bars: 10 µm.

Given the well-established association of AnxA2 with the endosomal system (Gerke et al., 2005; Futter and White, 2007; Grieve et al., 2012), we subjected NRK cells to long-term uptake of fluorescent transferrin (Tfn), which allows for the visualization of endocytic membrane compartments at various stages of the cell cycle (Sager et al., 1984; Tacheva-Grigorova et al., 2013; Takatsu et al., 2013). As previously shown for interphase NRK cells (Marie et al., 2009), internalized transferrin labels both the peripheral endosomes and pericentrosomal REs. Notably, the AnxA2-containing structures in mitotic NRK cells also contained fluorescent Tfn (Figures 2A–C), indicating their connection to the endosomal system. Additionally, these structures were positive to the transferrin receptor (data not shown), and the GTPase Rab11 (Supplementary Figure S4; panels D-F), another commonly used marker of REs. This suggests that the association of AnxA2 with the endocytic recycling apparatus (Zeuschner et al., 2001; Zobiack et al., 2003; Delevoye et al., 2016) is maintained throughout mitosis.

Furthermore, since the pre-Golgi IC and REs–identified by the GTPases Rab1 and Rab11, respectively–maintain their connection during mitosis (Marie et al., 2012), we sought to re-examine the localization of Rab1 in mitotic NRK cells fixed with PLP. Notably, the use of cells expressing green fluorescent protein (GFP)-tagged Rab1, as well as antibodies targeting the endogenous protein, both revealed the presence of Rab1 in the mitotic structures containing AnxA2 or transferrin (Figures 2D–F). It should be noted that while the PLP-fixation aids in visualizing endogenous Rab1 in the peripheral mitotic structures, it diminishes the strong signal of the protein in the IC elements located at the spindle poles (Marie et al., 2012).

We also investigated the presence of other commonly used organelle markers in the novel mitotic structures. Regarding organelles of the secretory pathway (Supplementary Figure S2), antibodies against the ER resident protein calnexin (panels A-C) and proteins localized to the *cis*, *medial-* and *trans*-Golgi compartments–specifically, GM130 (panels D-F), mannosidase II (panels G-I), and TGN46 (panels J-L), respectively–failed to detect any of these proteins in the mitotic structures. Furthermore, treatment of cells with the drug brefeldin A (BFA), which quickly disrupts membrane-bound coat protein I (COPI) coats and leads to extensive Golgi disassembly, did not significantly affect these structures. This suggests that the Golgi apparatus and the COPI machinery do not play a role in their formation or maintenance (Supplementary Figure S3). Finally, as shown in Supplementary Figure S4, double staining of cells with antibodies against Rab7 (panels A-C), EEA1 (panels G-I), or LAMP-1 (panels J-L) indicated that the structures are also devoid of early or late endosomes, as well as lysosomes.

Since AnxA2 has been implicated in actin dynamics (Hayes et al., 2006; Rescher et al., 2008; Hayes et al., 2009; Grieve et al., 2012), it is possible that the observed mitotic structures contained aggregates of actin filaments. However, the F-actin probe phalloidin was not detected in these structures (Supplementary Figure S5), and the integrity of the AnxA2-containing structures was preserved in cells treated with the actin filament depolymerizing drug latrunculin B (data not shown). Alternatively, these structures could be related to nuclear promyelocytic leukaemia (PML) bodies, which contain Tyr23 phosphorylated AnxA2 (Grindheim, et al., 2016). During mitosis, these bodies are released into the cytoplasm and associate with early endosomes marked by the early endosomal antigen 1 (EEA1) (Palibrk et al., 2014). However, this possibility was ruled out due to the absence of the PML protein in the AnxA2-positive mitotic structures (data not shown). Finally, the regular spacing of the structures raised the possibility of their association with the ERM proteins (ezrin, radixin and moesin) which link actin filaments and MTs to the PM (Vilmos et al., 2016). However, the staining patterns of antibodies against moesin or phosphorylated ERM proteins (anti-pERM), which co-align with the PM, were distinct from the AnxA2-positive spherical structures, which are located at a distance from the PM (Supplementary Figure S5).

In conclusion, these results reveal a specific connection between the AnxA2-containing mitotic structures and the tubular networks involved in membrane recycling at the ER-Golgi boundary (IC) or within the endosomal system (REs).

### 2.3 The mitotic structures co-align with astral MTs

**FIGURE 3:**
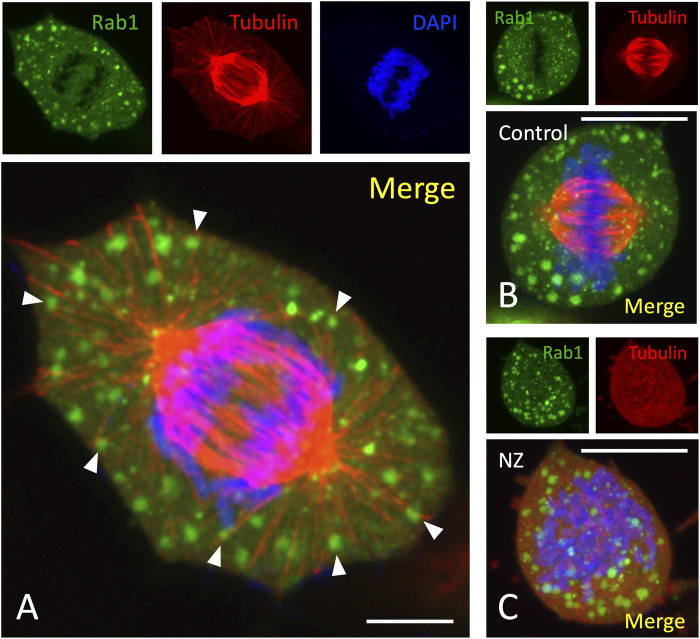
*Apparent association of the mitotic structures with astral MTs*. NRK cells expressing GFP-Rab1 were fixed with PLP and stained with monoclonal antibodies against β-tubulin and DAPI. **(A)** shows an image of a cell at early anaphase, suggesting possible co-localization of the large mitotic structures with the astral MTs radiating from the spindle poles (white arrowheads). Note also the absence of these structures from the spindle area. **(B,C)** show representative images of similarly stained control metaphase cells **(B)**, and cells treated for 30 min with nocodazole (NZ) to disassemble the spindle MTs **(C)**. Note the dispersal of the large mitotic structures in the drug-treated cells. Scale bars: 5 µm **(A)** or 10 µm **(B,C)**.

**FIGURE 4:**
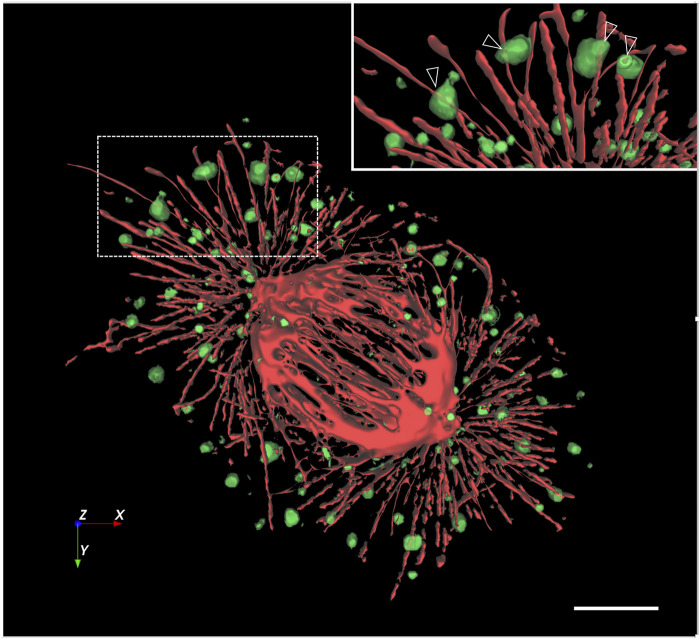
*Image processing shows co-alignment of the mitotic structures with astral MTs.* Z-stacks of the cell shown in Figure 3A were processed by volumetric segmentation and 3D rendering, revealing a close connection between the large peripheral structures–which were made partly transparent–and astral MTs, as highlighted in the inset (open arrowheads). Scale bar: 5 µm.

The preferential localization of the mitotic structures to the cell periphery raises the possibility that they are related to the conserved protein complexes that mediate the cortical anchoring of astral MTs and have recently been shown to contain AnxA2 (Di Pietro et al., 2016; Pascal et al., 2022). Comparison of the distributions of the Rab1-containing mitotic structures and β-tubulin-containing MTs in anaphase cells showed that the former are absent from the region of the central mitotic spindle. However, many of the large peripheral structures (ca. 75%) reside in close vicinity of the astral MTs radiating from the spindle poles (Figure 3A, arrowheads), as further demonstrated by 3D image reconstructions (Figure 4). Notably, our previous studies have already shown that the Rab1-positive IC elements are preserved and maintain their association with the spindle MTs during mitosis (Marie et al., 2012).

To obtain additional information on the functional relationship of the peripheral structures with the mitotic spindle, cells were treated with nocodazole (NZ) to find out whether their structure and/or localization is affected by the disassembly of the spindle MTs. Interestingly, whereas the spherical shape of the structures is unaffected by MT depolymerization, they assume a more dispersed distribution in the drug-treated cells (Figures 3B,C). Thus, despite their apparent association with astral MTs, these assemblies are structurally independent of the spindle which, however, may influence their cellular localization.

### 2.4 The mitotic structures have properties of biomolecular condensates

The large size and spherical shape of the mitotic structures, as well as the need to introduce specific fixation protocols to improve their structural preservation suggested that they may represent biomolecular condensates (Banani et al., 2017), rather than classical membrane-bound organelles. To explore this possibility, we tested their response to aliphatic alcohols, 1,6-hexanediol and 1,2-propanediol–also known as propylene glycol (PG) – which selectively dissolve cellular assemblies formed via liquid-liquid phase separation (LLPS) (Kroschwald et al., 2017; Geiger et al., 2021). However, consistent with its known toxicity (Kroschwald et al., 2017), 1,6-hexanediol dramatically altered the morphology of mitotic NRK cells, causing many metaphase cells to collapse (data not shown). By contrast, the non-toxic PG is well tolerated by mammalian cells (Mochida and Gomyoda, 1987; Geiger et al., 2021) and proved to be applicable also for the investigation of mitotic cells.

**FIGURE 5:**
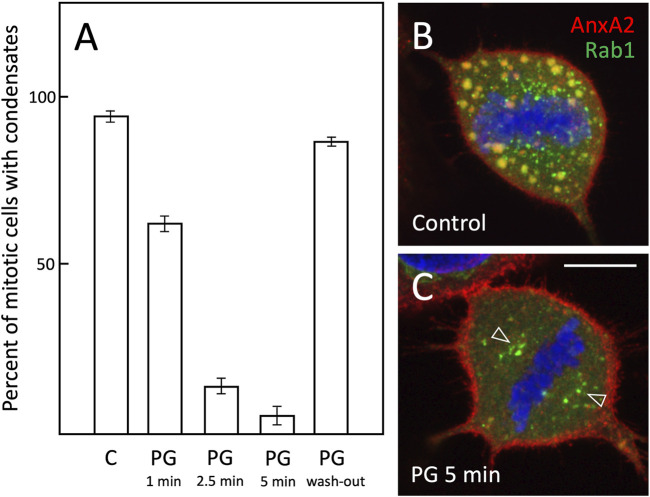
*The mitotic structures have properties of biomolecular condensates*. **(A)** NRK cells expressing GFP-Rab1 were left untreated (Control), treated for 1–5 min with propylene glycol (PG), or treated for 5 min with PG, followed by a 30 min wash-out of the aliphatic alcohol. After fixation and staining for AnxA2 and DAPI the percentage of cells at meta- or anaphase, containing structures positive for both Rab1-and AnxA2 were determined. Average values derived from the analysis of three independent samples and the standard deviations (SD) are shown. The average number of cells analysed was as follows: Control (n = 107), PG 1 min (n = 96), PG 2.5 min (n = 79), PG 5 min (n = 66); PG washout (n = 80). **(B,C)** show representative images of control metaphase cells and cells treated for 5 min with PG, respectively. Note breakdown of the large peripheral structures, increased cortical AnxA2 signal and preservation of the spindle pole-associated Rab1-positive IC elements (open arrows) in the PG-treated cells. Scale bar: 10 µm.

**FIGURE 6:**
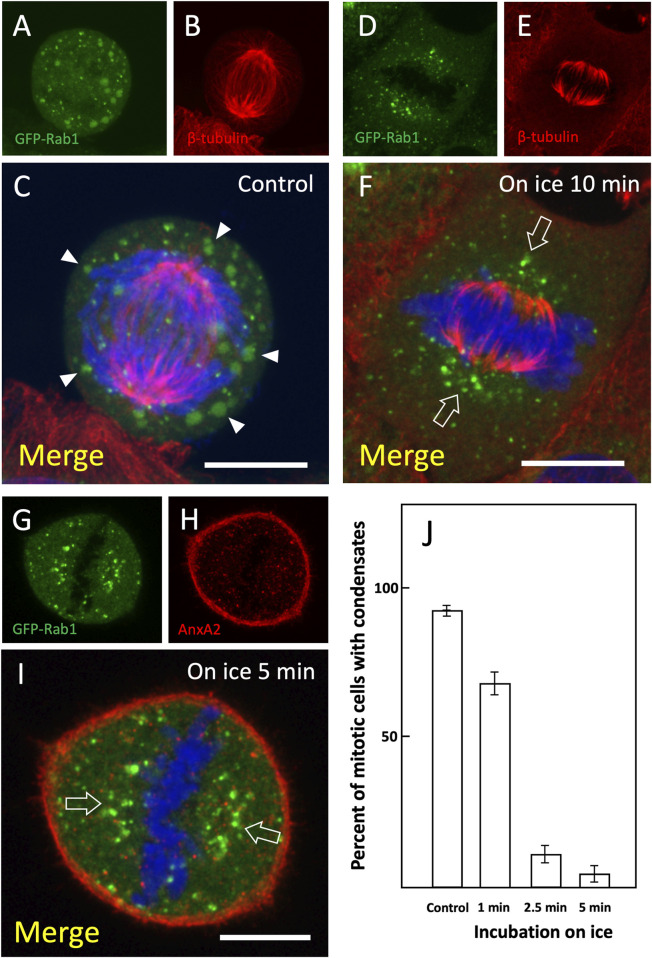
*Disassembly of the mitotic condensates at low temperature.* Control GFP-Rab1 expressing cells **(A–C)** and cells incubated for variable times in ice-cold culture medium prior to fixation were stained for β-tubulin **(D–F)** or AnxA2 **(G–J)**. Low temperature results in selective breakdown of the peripheral mitotic structures (C; arrowheads) without affecting the mitotic spindle **(F)** or the Rab1-positive IC elements at the spindle poles [**(F,I)**, open arrows]. **(J)** Quantification of the effect of low temperature on the peripheral structures positive for both GFP-Rab1 and AnxA2 was carried out as described for Figure 5A. The average number of cells analysed was as follows: Control (n = 111), on ice incubation 1 min (n = 131), 2.5 min (n = 87), and 5 min (n = 82); Scale bars: 10 µm.

Quantitation showed that the addition of PG even at relatively low concentration (2.5%) rapidly reduced the number of meta- and anaphase cells containing the mitotic structures and by 5 min they had almost completely disappeared (Figure 5A). Following the removal of PG for 30 min the percentage of positive cells returned to the initial control level, showing that its cellular effects are readily reversible (Figure 5A). Microscopy of control and PG-treated metaphase cells showed that the break-down of the AnxA2-and Rab1-containing large structures by PG is not accompanied by major changes in cell shape or misalignment of the chromosomes at the equatorial plane (Figures 5B,C). Moreover, PG did not affect the accumulation of the Rab1-positive IC membranes at the spindle poles (Marie et al., 2012); Figure 5C, arrowheads), further indicating that the mitotic spindle is not affected. Interestingly, brief incubation of cells at low temperature – possibly by affecting membrane fluidity and/or protein-lipid interactions–gave similar results to those obtained with PG (Figure 6). Accordingly, in response to 1–5 min incubation of cells in ice-cold culture medium, the peripheral mitotic structures gradually disappeared, while the MT-based mitotic spindle and Rab1-positive IC elements at the spindle poles appear to remain unaffected (Figure 6).

In conclusion, the selective breakdown of the large mitotic structures by PG or low temperature strongly suggests that they correspond to biomolecular condensates rather than conventional membrane-bound organelles.

### 2.5 The mitotic condensates contain lamin B

Biomolecular condensation via LLPS has also been implicated in the assembly of mitotic structures, including the expansion of the pericentriolar material (PCM) and the formation of the spindle matrix – a membranous assembly of nuclear proteins that is independent of MTs but functionally linked to the mitotic spindle (Woodruff, 2018; Tiwary and Zheng, 2019). As noted in the Introduction, lamin B–a component used to define the spindle matrix (Tsai et al., 2006; Ma et al., 2009) – is released as the nuclear lamina breaks down during prometaphase but remains connected to membranes (Champion et al., 2017). Since the novel mitotic structures described in this study exhibit properties of membrane-associated biomolecular condensates, it was of interest to investigate whether they contain lamin B.

**FIGURE 7:**
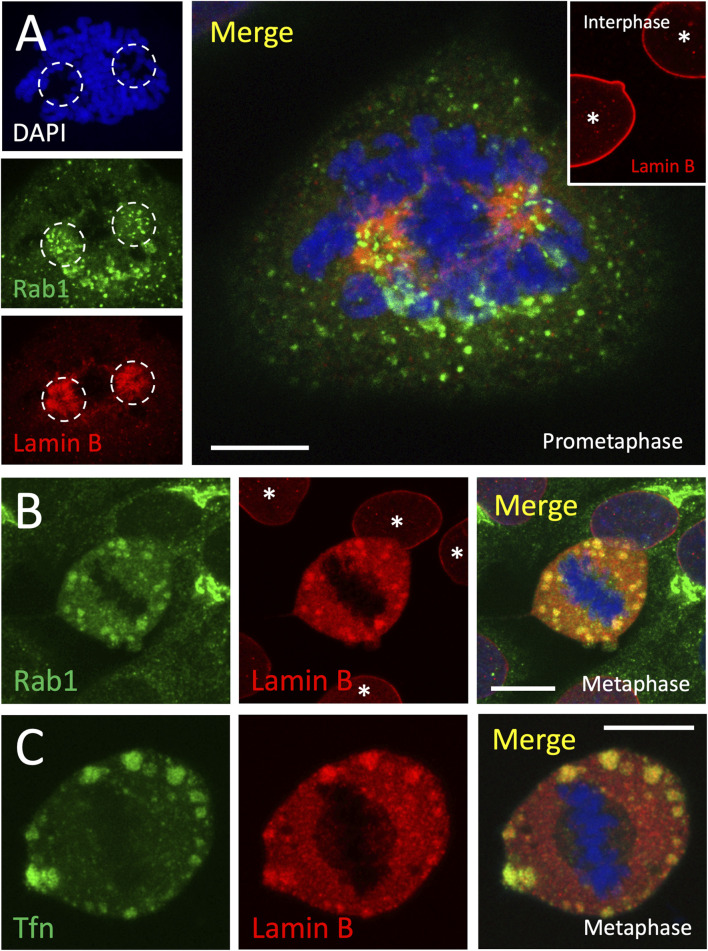
*The mitotic structures contain lamin B.* NRK cells expressing GFP-Rab1 **(A,B)**, or the corresponding parental NRK cells subjected to uptake of Alexa-Fluor 488 Tfn **(C)** were fixed and stained for lamin B (red) and DAPI (blue). **(A)** shows a cell at prometaphase, in which lamin B – released from the disassembled nuclear lamina–and the Rab1-positive pericentrosomal IC elements partly colocalize at the separating spindle poles residing in nuclear invaginations revealed by the DAPI-staining (dashed circles). The inset shows lamin B staining of interphase cells (asterisks). B and C demonstrate the co-localization of lamin B with Rab1 and Tfn in the large mitotic condensates at the periphery of metaphase cells. Note the additional diffuse cytoplasmic staining for lamin B and its absence–shown by appropriate projections **(C)** – from the spindle area. Scale bars: 5 µm **(A)**, 10 µm **(B,C)**.

Indeed, double localization of lamin B in PLP-fixed metaphase cells with Rab1, endocytosed transferrin (Tfn) or Rab11 demonstrated its extensive overlap with these markers of membrane recycling compartments that associate with the mitotic condensates (Figures 7B,C; Supplementary Figure S4; panels D–F), raising the possibility that they are related to the lamin B-containing membranous spindle matrix (Zheng, 2010). Besides staining large peripheral mitotic structures, the lamin B antibodies gave diffuse cytoplasmic signal which was absent from the spindle region (Figure 7C).

To gain insight into the pathway by which lamin B reaches the peripheral condensates, we examined cells at prometaphase, when the NE and the nuclear lamina break down in a process tightly coupled to centrosome separation and spindle formation (Champion et al., 2017). Previous studies of NRK cells have shown that at mitotic onset both lamin B (Beaudouin et al., 2002) and the pericentrosomal membrane recycling compartments, defined by Rab1 and Rab11 (Marie et al., 2012), accumulate around the forming spindle poles. Indeed, at the time when the separating centrosomes localize to deep NE invaginations (Robbins and Gonatas, 1964; Beaudouin et al., 2002; Salina et al., 2002), lamin B and the Rab1-positive IC elements concurrently accumulate around the spindle poles, displaying overlapping distributions (Figure 7A). This finding suggests that the transfer of lamin B from the disassembling nuclear lamina to the large peripheral condensates emerging at prometaphase occurs via its association with the membrane recycling compartment(s) at the spindle poles.

### 2.6 Equal partitioning of the mitotic condensates

**FIGURE 8:**
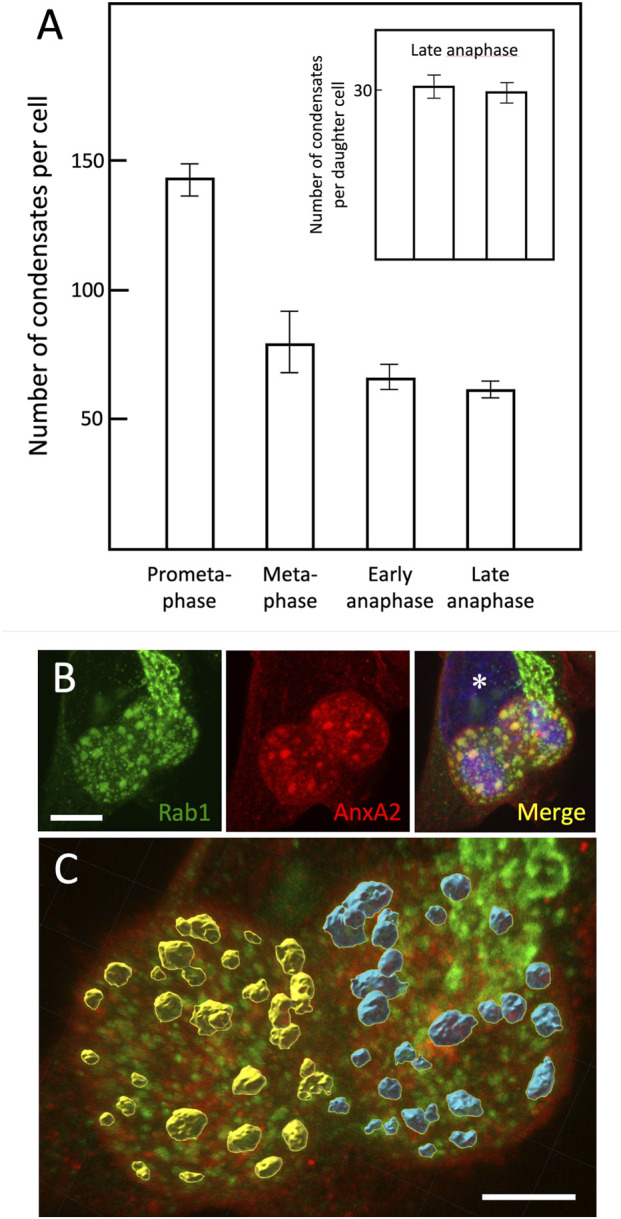
*Equal partitioning of the condensates during mitosis*. **(A)** Following fixation and staining of GFP-Rab1-expressing cells for AnxA2, the number of the condensates in cells at different phases of mitosis was determined. The average number of condensates in prometaphase (n = 4), metaphase (n = 7), early anaphase (n = 5) late anaphase cells (n = 13), as well as the standard deviations (SD) are shown. The inset shows similar quantitation carried out for the separating daughter cells at late anaphase. **(B,C)** show representative images of a late anaphase cell, in which the borders of the daughter cells can be readily identified due to the constriction created by the developing cleavage furrow. An interphase cell is marked by an asterisk. **(C)**, corresponding to Supplementary Movie-2, was generated by the Imaris software and illustrates the differentially pseudo-coloured (yellow or blue) large Rab1-and AnxA2-positive condensates in the two daughter cells. Scale bars: 10 µm **(B)**, 5 µm **(C)**.

As discussed earlier, as cells progress through mitosis, the size of the condensates increases while their number appears to decrease (Figure 1). Quantification of the average number of condensates in cells at different mitotic stages–from prometaphase to late anaphase–revealed that their number drops by about half between prometaphase and metaphase–possibly based on their fusion–but then stabilizes during anaphase (Figure 8A). Notably, at late anaphase, the separating daughter cells, clearly delineated by the cleavage furrow (Figures 8B,C; Supplementary Figure S6), contain almost equal numbers of the condensates (Figure 8A; inset; see also Supplementary Movie-2). The estimated total volumes of the two pools of condensates were also similar – 34.8 vs. 32.9 µm^3^ for the cell shown in Figure 8C; Supplementary Movie-2) – indicating that their partitioning is a tightly regulated process.

## 3 Discussion

The present investigation utilizing specific fixation protocols to determine the localization of AnxA2 during cell division, led to identification of novel structures that transiently appear at the periphery of mitotic cells. The exceptionally large size and spherical shape of these structures, along with their apparent ability to fuse, are characteristic features of biomolecular condensates formed through phase separation (Banani et al., 2017). Furthermore, the rapid and selective breakdown of these structures by the aliphatic alcohol propylene glycol (PG), known to disrupt weak hydrophobic protein-protein and protein-RNA interactions (Geiger et al., 2021), as well as low temperature, supports the conclusion that they represent biomolecular condensates rather than classical membrane-bound organelles. In contrast to the typically membraneless cytoplasmic biomolecular condensates, such as P-bodies and stress granules (Banani et al., 2017), the mitotic condensates identified in this study contain specific membranes. In this respect they bear resemblance, for instance, to the protein condensates operating in vesicle trafficking at the ER-Golgi boundary or in the presynaptic regions of neurons, the latter enclosing distinct subpopulations of synaptic vesicles that closely associate with the PM (Qiu et al., 2024; Ruiz et al., 2026).

Interestingly, these mitotic structures exhibit similarities to the spindle matrix, an enigmatic assembly of nuclear proteins–including the nuclear lamina subunit lamin B–whose formation has also been suggested to involve phase separation (Schweizer et al., 2014; Jiang et al., 2015; Woodruff, 2018; Tiwary and Zheng, 2019). Particularly, the presence of lamin B and the two membrane compartments in these structures suggest their relationship to the previously proposed membranous (detergent-sensitive) lamin B spindle matrix. This structure is thought to surround and mechanically support the MT-based spindle, thereby regulating its proper assembly, orientation and function (Zheng, 2010; Johansen et al., 2011; Schweizer et al., 2014; Scholey, 2025).

Notably, information regarding the mitotic roles of the protein components identified in the present study further emphasizes the similarity of the condensates to the spindle matrix. By interacting with actin and PI(4,5)P_2_ (Harder et al., 1997; Rescher et al., 2004; Gerke et al., 2005; Hayes et al., 2009), AnxA2 exhibits properties characteristic of a protein involved in locking of astral MTs to regularly spaced foci at the cell periphery (Sandquist et al., 2011). Indeed, it was recently shown to collaborate with Ahnak, NuMA and the MT-based motor protein dynein in the cortical anchoring of astral MTs, thereby facilitating spindle positioning (Pascal et al., 2022). Given that the assembly of biomolecular condensates can be regulated by phosphorylation of their key protein components (Sridharan et al., 2022), it will be of interest to determine the phosphorylation status of AnxA2 in mitotic vs. interphase cells.

Regarding lamin B, earlier studies by Zheng and colleagues demonstrated its interaction with various spindle assembly factors, including NuMA and the motor proteins dynein and kinesin, Eg5 (Tsai et al., 2006; Ma et al., 2009). Furthermore, the GTPase Rab11 has been shown to maintain its association with REs during mitosis (Hobdy-Henderson et al., 2003; Marie et al., 2012; Hehnly and Doxsey, 2014) and to regulate dynein-dependent delivery of key PCM components, such as γ-tubulin and pericentrin, to the spindle poles. These transport events promote the nucleation of astral MTs and ensure correct spindle orientation (Hehnly and Doxsey, 2014). Moreover, studies involving knock-out mice have demonstrated that the Rab11A and Rab11B isoforms redundantly regulate spindle function in dividing epithelial progenitor cells (Joseph et al., 2023). Finally, depletion of the IC-associated Rab1 has been reported to impact centrosome maturation and spindle assembly, as well as lead to endomembrane alterations in the mitotic cells of the *Drosophila* embryos (Rollins and Blankenship, 2023).

The extensive reorganization of endomembranes that occurs during mitosis has traditionally been thought to coincide with the inhibition of membrane traffic (Shorter and Warren, 2002; Fielding and Royle, 2013). However, more recent studies indicate that endocytosis continues throughout mitosis, although the rate of endocytic uptake–possibly via different pathways–appears to slow down from prometaphase to anaphase (Tacheva-Grigorova et al., 2013; Aguet et al., 2016; Hinze and Boucrot, 2018). In contrast, it has been proposed that endocytic membrane recycling back to the PM is temporarily arrested between prophase and late anaphase, providing a straightforward mechanism for mitotic regulation of cell surface area (Boucrot and Kirchhausen, 2007; Devenport et al., 2011). It is possible that the mitotic effects on different steps of membrane traffic are closely related to the development of the peripheral condensates into repositories for the biosynthetic (IC) and endocytic (RE) tubular networks that also converge at the spindle poles (Marie et al., 2012; Saraste and Marie, 2018) (Figure 7). Upon their disassembly at telophase, the condensates could serve as a membrane source for the expansion of the cell surface taking place at the intercellular bridge, explaining the localization of both AnxA2 and Rab11 at this site (Skop et al., 2004; Takahashi et al., 2011).

**FIGURE 9:**
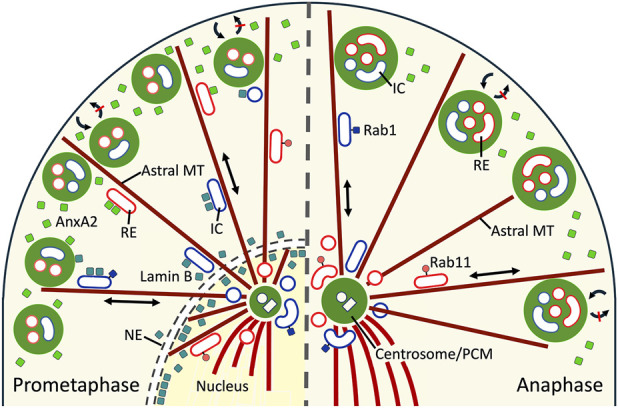
*Schematic model on the events proposed to take place in the biogenesis of the AnxA2-and lamin B-containing membranous mitotic condensates*. The illustrated sections of prometaphase (left) and anaphase (right) cells, separated by a dashed line, include only one of the spindle poles. At prometaphase the emergence of the AnxA2-and lamin B-containing peripheral condensates, depicted by green spheroids, coincides with the breakdown of the nuclear envelope (NE) and lamina. The released lamin B is proposed to move from the spindle poles to the peripheral condensates bound to Rab1-positive IC elements (as shown here) or Rab11-positive REs, based on their motor-dependent trafficking along astral MTs. The recruitment of AnxA2 to the condensates may occur from the cortical cytoplasm or involve its association with the REs. The accumulation of the membrane recycling compartments in the condensates could be due to their proposed communication with the pericentrosomal IC elements and REs at the spindle poles. In addition, the pile-up of REs could result from ongoing endocytic traffic from the cell surface, combined with inhibition of membrane recycling. Two-way communication between the growing condensates and the spindle poles may also be linked to the expansion of the pericentrosomal material (PCM), which has been proposed to involve phase separation.

The discovery of mitotic functions for selected Rab proteins–commonly regarded as master regulators of membrane traffic–has revealed that certain transport steps remain largely unaffected during mitosis (Capalbo et al., 2011; Serio et al., 2011; Miserey-Lenkei and Colombo, 2016). Together with the above mentioned observations regarding Rab11 (Hehnly and Doxsey, 2014), the present results suggest the possibility of ongoing motor-dependent transport along the astral MTs that connect the peripheral condensates with the spindle poles, as illustrated in the model shown in Figure 9. Indeed, the presence of the biosynthetic and endocytic membrane recycling compartments at both the peripheral condensates and the spindle poles supports the idea that these sites communicate via membrane traffic (Figure 9). Given that phase separation has been implicated in both the assembly of the spindle matrix and the expansion of the PCM (Woodruff, 2018; Tiwary and Zheng, 2019), it is tempting to speculate that the events occurring at the plus and minus ends of astral MTs may involve the exchange of key components (Figure 9). For instance, NuMA, which associates with AnxA2 and lamin B, and is found at both the cell cortex and the spindle poles, regulates spindle assembly and function through phase separation (Ma et al., 2009; Sun et al., 2021). Furthermore, lamin B is expected to utilize the astral MTs as tracks for its transfer from the spindle poles to the peripheral condensates as the nuclear lamina disassembles at prometaphase (Figure 9).

In interphase, local concentration of proteins and nucleic acids created by biomolecular condensation gives rise to organelles with distinct synthetic activities and functions (Banani et al., 2017). Since cellular processes slow down during mitosis, concentrating cell constituents via phase separation could also facilitate their accurate partitioning. Indeed, the finding that the forming daughter cells at late anaphase contain equal numbers of the condensates supports their role in the partitioning process. The presence of lamin B in these structures suggests that they contribute to the mitotic segregation of nuclear proteins. Additionally, the role of AnxA2 as an mRNA-binding protein that regulates the transport and translation of specific mRNAs (Vedeler and Hollas, 2000; Mickleburgh et al., 2005; Vedeler et al., 2012; Grindheim et al., 2016; Grindheim et al., 2017; Strand et al., 2021; Grindheim et al., 2023) raises the possibility that the condensates participate in the partitioning of selected mRNAs. Finally, the identification of the biosynthetic and endocytic membrane recycling compartments as components of the mitotic condensates suggests a role for phase separation in the division of endomembranes.

## 4 Materials and methods

### 4.1 Antibodies and reagents

Mouse monoclonal antibodies against AnxA2 (1:200 dilution), early endosomal antigen 1 (14/EEA1; 1:50 dilution), Rab11 (47/Rab11; 1:50 dilution), GM130 (35/GM130; 1:200 dilution) and TGN38 (2/TGN38; 1:50 dilution) were purchased from BD Transduction Laboratories. Rabbit polyclonal antibodies against AnxA2 (ab41803; 1:250 dilution) and Lamin B (ab16048; 1:200 dilution) were obtained from Abcam. The mouse monoclonal transferrin receptor antibody (H68.4; 1:200 dilution) and rabbit polyclonal Rab11 antibody (71–5300; 1:50 dilution) were from Invitrogen, while the mouse monoclonal antibody against LAMP-1 (H5G11; 1:200 dilution) and rabbit polyclonal antibody against Rab7 (R4779; 1:50 dilution) were purchased from Santa Cruz Biotechnology and Sigma, respectively. The rabbit monoclonal antibodies against phosphorylated ERM proteins (p-ERM; #3141; 1:100 dilution) and moesin (ab52490; 1:200 dilution) were bought from Cell Signaling Technology and Abcam, respectively. The mouse monoclonal antibody against Rab1B (1:200 dilution) was generously provided by Angelica Barnekow (University of Münster, Germany), while the rabbit polyclonal antibodies against calnexin (1:100 dilution) and mannosidase II (1:500 dilution) were generous gifts from Ari Helenius (ETH, Zürich, Switzerland) and Kelley Moremen (University of Georgia, United States), respectively. The mouse ascites fluid against β-tubulin (T13; 1:500 dilution) was provided by the late Thomas Kreis. The primary antibodies were detected using appropriate Alexa Fluor 488- or Alexa Fluor 594-conjugated secondary goat anti-mouse or anti-rabbit Fab_2_-fragments (1:100 dilution) bought from Jackson Immuno-Research Laboratories. Alexa Fluor 488- and Alexa Fluor 594-conjugated human transferrin and Alexa Fluor 594-conjugated phalloidin were obtained from Invitrogen. Brefeldin A (BFA), nocodazole (NZ) and propylene glycol (PG) were purchased from Sigma.

### 4.2 Cell culture

Normal rat kidney (NRK) cells and NRK cells stably expressing the GFP-Rab1 fusion protein (Marie et al., 2009) were grown in Dulbecco’s Minimum Essential Medium (DMEM) supplemented with 10% heat-inactivated fetal calf serum (FCS), 2 mM L-glutamine, 50 units/mL penicillin and 50 μg/mL streptomycin. Mitotic synchronization with drugs was not employed to avoid possible secondary effects. To obtain steady state cultures with high mitotic index, 100% confluent cultures of cells were diluted 1:2 and plated on 18 mm diameter glass coverslips in 6-well plates, followed by growth for 22–24 h. Baby hamster kidney (BHK21) cells, human HeLa cells, human retinal pigment epithelial (RPE-1) cells and rat embryo fibroblasts (REF52) were cultured as described elsewhere (Palokangas et al., 1998; Marie et al., 2012).

### 4.3 Transferrin uptake

To obtain fluorescent labeling of the endocytic membrane recycling compartments via uptake of transferrin the cells were first washed twice with prewarmed 37 °C DMEM supplemented with 0.2% bovine serum albumin (BSA) and 10 mM HEPES, pH 7.2, followed by incubation for 60 min at 37 °C in the same serum-free medium. For transferrin uptake, cells were incubated for an additional 60 min in the serum-free medium containing 20 μg/mL of human transferrin coupled to Alexa Fluor 488 or Alexa Fluor 594.

### 4.4 Experimental treatments

To release membrane-bound COPI coats and induce complete breakdown of the Golgi apparatus, NRK cells were incubated for 30 min at 37 °C in medium containing 5 μg/mL BFA. The disassembly of MTs and the spindle apparatus was obtained by 30 min treatment with 10 μg/mL nocodazole (NZ). To selectively disassemble biomolecular condensates, the cells were incubated for 1, 2.5 or 5 min at 37 °C in culture medium supplemented with 2.5% propylene glycol (PG). Upon wash-out the cells were washed twice with 37 °C culture medium, followed by incubation for an additional 30 min in the absence of PG. When the effect of low temperature on the mitotic condensates was examined, the coverslips were immersed for 1, 2.5 or 5 min in ice-cold culture medium prior to fixation.

### 4.5 Fixation conditions

In the standard sample preparation protocol the cells grown on glass coverslips were fixed for 60 min with 3% paraformaldehyde (PFA) in 0.1 M Na-phosphate buffer (pH 7.2) at RT. To obtain better structural preservation of the mitotic condensates the cells were fixed for 120 min with ice-cold PFA-lysine-sodium periodate (PLP) fixative (McLean and Nakane, 1974), consisting of 2% PFA, 0.075 M lysine-HCl and 0.01 M NaIO_4_ in 0.375 M Na-phosphate buffer, pH 6.2). This fixative preserves cell morphology better by cross-linking carbohydrates, retaining at the same time the antigenicity of proteins (Brown and Farquhar, 1989). The coverslips were quickly immersed in ice-cold fixative and kept on ice for the first 30 min, followed by transfer to RT for the remaining 90 min. In some experiments cells were further fixed and permeabilized by incubation for 5 min at −20 °C in ice-cold methanol.

### 4.6 Immunofluorescence staining, confocal microscopy and image processing

In most experiments, the fixed cells were permeabilized using 0.2% saponin with saponin being present throughout the labelling protocol. The immunofluorescence staining protocol, including the exposure of antigenic sites using guanidine-HCl (Peränen et al., 1993) has been described in detail previously (Sannerud et al., 2008; Raddum et al., 2013). After staining, the cells were first examined in a Zeiss Axiovert 200M inverted microscope equipped with long-working distance Plan-NEOFLUAR ×40 and ×100 objectives and fluorescence filter appropriate for Alexa 488, Alexa 594 and DAPI. Confocal microscopy on selected specimens was carried out to obtain individual optical sections or Z-stacks (step size 0.3 or 0.5 μm) using a Leica SP5 AOBS or Leica TCS SP8 confocal laser scanning microscopes, equipped with a 63x/1.4 NA Plan-Apochromat or 100x NA1.4 HC PL APO STED White oil-immersion objectives, 1 Airy unit pinhole aperture, 405 Diode, Argon and Helium-Neon lasers and the appropriate filter combinations. The images prepared using ImageJ are presented as single sections or maximum-intensity projections. Imaris software was used for image processing and preparation of the animations (see Supplementary Movies-1, 2).

For the volumetric segmentation of astral MTs and the large Rab1-positive mitotic structures, we used Aivia software (Leica Microsystems). Pixel classification was applied to identify the regions of interest in the red and green channels, serving as the basis for training of the software and subsequent segmentation of the two types of structures. To facilitate visualization of the possible vicinity of red structures with the green ones, the latter were rendered with 45% transparency (Figure 4).

### 4.7 Quantifications

The Zeiss Axiovert 200M fluorescence microscope equipped with the ×100 objective was used to determine the percentages of cells at different phases of mitosis or cytokinesis, positive for the large AnxA2-positive structures, by examining a total of 600 mitotic cells (Figure 1B). The same instrument was employed for the determination of the effects of the aliphatic alcohol PG (Figure 5A) and low temperature (Figure 6K) on the large mitotic structures, based on the examination of 66–131 meta- or anaphase cells for each experimental condition. Average values from three independent experiments and the standard deviations (SD) were determined. To establish the average size of the mitotic condensates in prometa-, meta- and anaphase cells (Figure 1C), Z-stacks were generated by the Leica TCS SP8 confocal microscope, followed by measurement of the diameters of up to 50 structures for each mitotic stage from maximum intensity projections corresponding to the center of the cell. The numbers of cells subjected to confocal optical sectioning to calculate the approximate number of the large condensates in cells at different stages of mitosis (Figure 8A) were as follows: prometaphase (n = 4), metaphase (n = 7), early anaphase (n = 5) and late anaphase (n = 13).

## Data Availability

The raw data supporting the conclusions of this article will be made available by the authors, without undue reservation.

## References

[B1] Aguet F. Upadhyayula S. Gaudin R. Chou Y. Y. Cocucci E. He K. (2016). Membrane dynamics of dividing cells imaged by lattice light-sheet microscopy. Mol. Biol. Cell 27 (22), 3418–3435. 10.1091/mbc.E16-03-0164 27535432 PMC5221578

[B2] Ayala I. Mascanzoni F. Colanzi A. (2020). The Golgi ribbon: mechanisms of maintenance and disassembly during the cell cycle. Biochem. Soc. Trans. 48 (1), 245–256. 10.1042/BST20190646 32010930

[B3] Banani S. F. Lee H. O. Hyman A. A. Rosen M. K. (2017). Biomolecular condensates: organizers of cellular biochemistry. Nat. Rev. Mol. Cell Biol. 18 (5), 285–298. 10.1038/nrm.2017.7 28225081 PMC7434221

[B4] Beaudouin J. Gerlich D. Daigle N. Eils R. Ellenberg J. (2002). Nuclear envelope breakdown proceeds by microtubule-induced tearing of the lamina. Cell 108 (1), 83–96. 10.1016/s0092-8674(01)00627-4 11792323

[B5] Benaud C. Prigent C. (2016). Annexin A2: a new player in mitosis. Cell Cycle 15 (1), 9–10. 10.1080/15384101.2015.1115643 26588095 PMC4825766

[B6] Benaud C. Le Dez G. Mironov S. Galli F. Reboutier D. Prigent C. (2015). Annexin A2 is required for the early steps of cytokinesis. EMBO Rep. 16 (4), 481–489. 10.15252/embr.201440015 25712672 PMC4388614

[B7] Bharadwaj A. Bydoun M. Holloway R. Waisman D. (2013). Annexin A2 heterotetramer: structure and function. Int. J. Mol. Sci. 14 (3), 6259–6305. 10.3390/ijms14036259 23519104 PMC3634455

[B8] Boucrot E. Kirchhausen T. (2007). Endosomal recycling controls plasma membrane area during mitosis. Proc. Natl. Acad. Sci. U. S. A. 104 (19), 7939–7944. 10.1073/pnas.0702511104 17483462 PMC1876551

[B9] Brown W. J. Farquhar M. G. (1989). Immunoperoxidase methods for the localization of antigens in cultured cells and tissue sections by electron microscopy. Methods Cell Biol. 31, 553–569. 10.1016/s0091-679x(08)61626-x 2674632

[B10] Capalbo L. D'Avino P. P. Archambault V. Glover D. M. (2011). Rab5 GTPase controls chromosome alignment through Lamin disassembly and relocation of the NuMA-like protein Mud to the poles during mitosis. Proc. Natl. Acad. Sci. U. S. A. 108 (42), 17343–17348. 10.1073/pnas.1103720108 21987826 PMC3198372

[B11] Carlton J. G. Jones H. Eggert U. S. (2020). Membrane and organelle dynamics during cell division. Nat. Rev. Mol. Cell Biol. 21 (3), 151–166. 10.1038/s41580-019-0208-1 32034394

[B12] Champion L. Linder M. I. Kutay U. (2017). Cellular reorganization during mitotic entry. Trends Cell Biol. 27 (1), 26–41. 10.1016/j.tcb.2016.07.004 27528558

[B13] Delevoye C. Heiligenstein X. Ripoll L. Gilles-Marsens F. Dennis M. K. Linares R. A. (2016). BLOC-1 brings together the actin and microtubule cytoskeletons to generate recycling endosomes. Curr. Biol. 26 (1), 1–13. 10.1016/j.cub.2015.11.020 26725201 PMC4713302

[B14] Devenport D. Oristian D. Heller E. Fuchs E. (2011). Mitotic internalization of planar cell polarity proteins preserves tissue polarity. Nat. Cell Biol. 13 (8), 893–902. 10.1038/ncb2284 21743464 PMC3149741

[B15] di Pietro F. Echard A. Morin X. (2016). Regulation of mitotic spindle orientation: an integrated view. EMBO Rep. 17 (8), 1106–1130. 10.15252/embr.201642292 27432284 PMC4967962

[B16] Ellenberg J. Siggia E. D. Moreira J. E. Smith C. L. Presley J. F. Worman H. J. (1997). Nuclear membrane dynamics and reassembly in living cells: targeting of an inner nuclear membrane protein in interphase and mitosis. J. Cell Biol. 138 (6), 1193–1206. 10.1083/jcb.138.6.1193 9298976 PMC2132565

[B17] Fielding A. B. Royle S. J. (2013). Mitotic inhibition of clathrin-mediated endocytosis. Cell Mol. Life Sci. 70 (18), 3423–3433. 10.1007/s00018-012-1250-8 23307073 PMC3939358

[B18] Fielding A. B. Schonteich E. Matheson J. Wilson G. Yu X. Hickson G. R. (2005). Rab11-FIP3 and FIP4 interact with Arf6 and the exocyst to control membrane traffic in cytokinesis. EMBO J. 24 (19), 3389–3399. 10.1038/sj.emboj.7600803 16148947 PMC1276165

[B19] Futter C. E. White I. J. (2007). Annexins and endocytosis. Traffic 8 (8), 951–958. 10.1111/j.1600-0854.2007.00590.x 17547702

[B20] Geiger F. Acker J. Papa G. Wang X. Arter W. E. Saar K. L. (2021). Liquid-liquid phase separation underpins the formation of replication factories in rotaviruses. EMBO J. 40 (21), e107711. 10.15252/embj.2021107711 34524703 PMC8561643

[B21] Gerace L. Blum A. Blobel G. (1978). Immunocytochemical localization of the major polypeptides of the nuclear pore complex-lamina fraction. Interphase and mitotic distribution. J. Cell Biol. 79 (2 Pt 1), 546–566. 10.1083/jcb.79.2.546 102651 PMC2110258

[B22] Gerke V. Creutz C. E. Moss S. E. (2005). Annexins: linking Ca2+ signalling to membrane dynamics. Nat. Rev. Mol. Cell Biol. 6 (6), 449–461. 10.1038/nrm1661 15928709

[B23] Gerke V. Gavins F. N. E. Geisow M. Grewal T. Jaiswal J. K. Nylandsted J. (2024). Annexins-a family of proteins with distinctive tastes for cell signaling and membrane dynamics. Nat. Commun. 15 (1), 1574. 10.1038/s41467-024-45954-0 38383560 PMC10882027

[B24] Grieve A. G. Moss S. E. Hayes M. J. (2012). Annexin A2 at the interface of actin and membrane dynamics: a focus on its roles in endocytosis and cell polarization. Int. J. Cell Biol. 2012, 852430. 10.1155/2012/852430 22505935 PMC3296266

[B25] Grindheim A. K. Vedeler A. (2016). Extracellular vesicles released from cells exposed to reactive oxygen species increase annexin A2 expression and survival of target cells exposed to the same conditions. Commun. Integr. Biol. 9 (4), e1191715. 10.1080/19420889.2016.1191715 27574537 PMC4988444

[B26] Grindheim A. K. Hollas H. Raddum A. M. Saraste J. Vedeler A. (2016). Reactive oxygen species exert opposite effects on Tyr23 phosphorylation of the nuclear and cortical pools of annexin A2. J. Cell Sci. 129 (2), 314–328. 10.1242/jcs.173195 26644180 PMC4732284

[B27] Grindheim A. K. Saraste J. Vedeler A. (2017). Protein phosphorylation and its role in the regulation of Annexin A2 function. Biochim. Biophys. Acta Gen. Subj. 1861 (11 Pt A), 2515–2529. 10.1016/j.bbagen.2017.08.024 28867585

[B28] Grindheim A. K. Patil S. S. Nebigil C. G. Désaubry L. Vedeler A. (2023). The flavagline FL3 interferes with the association of Annexin A2 with the eIF4F initiation complex and transiently stimulates the translation of annexin A2 mRNA. Front. Cell Dev. Biol. 11, 1094941. 10.3389/fcell.2023.1094941 37250892 PMC10214161

[B29] Harder T. Kellner R. Parton R. G. Gruenberg J. (1997). Specific release of membrane-bound annexin II and cortical cytoskeletal elements by sequestration of membrane cholesterol. Mol. Biol. Cell 8 (3), 533–545. 10.1091/mbc.8.3.533 9188103 PMC276102

[B30] Hayes M. J. Merrifield C. J. Shao D. Ayala-Sanmartin J. Schorey C. D. S. Levine T. P. (2004a). Annexin 2 binding to phosphatidylinositol 4,5-bisphosphate on endocytic vesicles is regulated by the stress response pathway. J. Biol. Chem. 279 (14), 14157–14164. 10.1074/jbc.M313025200 14734570 PMC1351152

[B31] Hayes M. J. Rescher U. Gerke V. Moss S. E. (2004b). Annexin-actin interactions. Traffic 5 (8), 571–576. 10.1111/j.1600-0854.2004.00210.x 15260827

[B32] Hayes M. J. Shao D. Bailly M. Moss S. E. (2006). Regulation of actin dynamics by annexin 2. EMBO J. 25 (9), 1816–1826. 10.1038/sj.emboj.7601078 16601677 PMC1456940

[B33] Hayes M. J. Shao D.-M. Grieve A. Levine T. Bailly M. Moss S. E. (2009). Annexin A2 at the interface between F-actin and membranes enriched in phosphatidylinositol 4,5,-bisphosphate. Biochim. Biophys. Acta (BBA) - Mol. Cell Res. 1793 (6), 1086–1095. 10.1016/j.bbamcr.2008.10.007 19022301 PMC7611824

[B34] Hehnly H. Doxsey S. (2014). Rab11 endosomes contribute to mitotic spindle organization and orientation. Dev. Cell 28 (5), 497–507. 10.1016/j.devcel.2014.01.014 24561039 PMC4030695

[B35] Hinze C. Boucrot E. (2018). Endocytosis in proliferating, quiescent and terminally differentiated cells. J. Cell Sci. 131 (23), jcs216804. 10.1242/jcs216804 30504135

[B36] Hobdy-Henderson K. C. Hales C. M. Lapierre L. A. Cheney R. E. Goldenring J. R. (2003). Dynamics of the apical plasma membrane recycling system during cell division. Traffic 4 (10), 681–693. 10.1034/j.1600-0854.2003.00124.x 12956871

[B37] Jiang H. Wang S. Huang Y. He X. Cui H. Zhu X. (2015). Phase transition of spindle-associated protein regulate spindle apparatus assembly. Cell 163 (1), 108–122. 10.1016/j.cell.2015.08.010 26388440 PMC4607269

[B38] Johansen K. M. Forer A. Yao C. Girton J. Johansen J. (2011). Do nuclear envelope and intranuclear proteins reorganize during mitosis to form an elastic, hydrogel-like spindle matrix? Chromosome Res. 19 (3), 345–365. 10.1007/s10577-011-9187-6 21274615

[B39] Joseph I. Flores J. Farrell V. Davis J. Bianchi-Smak J. Feng Q. (2023). RAB11A and RAB11B control mitotic spindle function in intestinal epithelial progenitor cells. EMBO Rep. 24 (9), e56240. 10.15252/embr.202256240 37424454 PMC10481667

[B40] Kroschwald S. Maharana S. Alberti S. (2017). Hexanediol: a chemical probe to investigate the material properties of membrane-less compartments. Matters. 10.19185/matters.2017020000010

[B41] Kumar D. Lak B. Suntio T. Vihinen H. Belevich I. Viita T. (2021). RTN4B interacting protein FAM134C promotes ER membrane curvature and has a functional role in autophagy. Mol. Biol. Cell 32 (12), 1158–1170. 10.1091/mbc.E20-06-0409 33826365 PMC8351555

[B42] Lu L. Ladinsky M. S. Kirchhausen T. (2009). Cisternal organization of the endoplasmic reticulum during mitosis. Mol. Biol. Cell 20 (15), 3471–3480. 10.1091/mbc.e09-04-0327 19494040 PMC2719565

[B43] Ma L. Tsai M. Y. Wang S. Lu B. Chen R. Yates J. R. III (2009). Requirement for nudel and dynein for assembly of the lamin B spindle matrix. Nat. Cell Biol. 11 (3), 247–256. 10.1038/ncb1832 19198602 PMC2699591

[B44] Mall M. Walter T. Gorjánácz M. Davidson I. F. Nga Ly-Hartig T. B. Ellenberg J. (2012). Mitotic lamin disassembly is triggered by lipid-mediated signaling. J. Cell Biol. 198 (6), 981–990. 10.1083/jcb.201205103 22986494 PMC3444782

[B45] Marie M. Dale H. A. Sannerud R. Saraste J. (2009). The function of the intermediate compartment in pre-golgi trafficking involves its stable connection with the centrosome. Mol. Biol. Cell 20 (20), 4458–4470. 10.1091/mbc.e08-12-1229 19710425 PMC2762134

[B46] Marie M. Dale H. A. Kouprina N. Saraste J. (2012). Division of the intermediate compartment at the onset of mitosis provides a mechanism for golgi inheritance. J. Cell Sci. 125 (22), 5403–5416. 10.1242/jcs.108100 22946056

[B47] Mayran N. Parton R. G. Gruenberg J. (2003). Annexin II regulates multivesicular endosome biogenesis in the degradation pathway of animal cells. EMBO J. 22 (13), 3242–3253. 10.1093/emboj/cdg321 12839987 PMC165635

[B48] McLean I. W. Nakane P. K. (1974). Periodate-lysine-paraformaldehyde fixative. A new fixation for immunoelectron microscopy. J. Histochem Cytochem 22 (12), 1077–1083. 10.1177/22.12.1077 4374474

[B49] Mickleburgh I. Burtle B. Hollas H. Campbell G. Chrzanowska-Lightowlers Z. Vedeler A. (2005). Annexin A2 binds to the localization signal in the 3’ untranslated region of c-myc mRNA. FEBS J. 272 (2), 413–421. 10.1111/j.1742-4658.2004.04481.x 15654879

[B50] Miserey-Lenkei S. Colombo M. I. (2016). Small Rab GTPases regulate multiple steps of mitosis. Front. Cell Dev. Biol. 4, 2. 10.3389/fcell.2016.00002 26925400 PMC4756281

[B51] Mochida K. Gomyoda M. (1987). Toxicity of ethylene glycol, diethylene glycol, and propylene glycol to human cells in culture. Bull. Environ. Contam. Toxicol. 38 (1), 151–153. 10.1007/BF01606573 3814844

[B52] Palibrk V. Lång E. Lång A. Schink K. O. Rowe A. D. Bøe S. O. (2014). Promyelocytic leukemia bodies tether to early endosomes during mitosis. Cell Cycle 13 (11), 1749–1755. 10.4161/cc.28653 24675887 PMC4111721

[B53] Palokangas H. Ying M. Väänänen K. Saraste J. (1998). Retrograde transport from the pre-gGolgi intermediate compartment and the golgi complex is affected by the vacuolar H+-ATPase inhibitor bafilomycin A1. Mol. Biol. Cell 9 (12), 3561–3578. 10.1091/mbc.9.12.3561 9843588 PMC25677

[B54] Pascal A. Gallaud E. Giet R. Benaud C. (2022). Annexin A2 and Ahnak control cortical NuMA-dynein localization and mitotic spindle orientation. J. Cell Sci. 135 (9), 259344. 10.1242/jcs.259344 35362526

[B55] Peränen J. Rikkonen M. Kääriäinen L. (1993). A method for exposing hidden antigenic sites in paraformaldehyde-fixed cultured cells, applied to initially unreactive antibodies. J. Histochem Cytochem 41 (3), 447–454. 10.1177/41.3.8429208 8429208

[B56] Puhka M. Vihinen H. Joensuu M. Jokitalo E. (2007). Endoplasmic reticulum remains continuous and undergoes sheet-to-tubule transformation during cell division in mammalian cells. J. Cell Biol. 179 (5), 895–909. 10.1083/jcb.200705112 18056408 PMC2099207

[B57] Puhka M. Joensuu M. Vihinen H. Belevich I. Jokitalo E. (2012). Progressive sheet-to-tubule transformation is a general mechanism for endoplasmic reticulum partitioning in dividing mammalian cells. Mol. Biol. Cell 23 (13), 2424–2432. 10.1091/mbc.E10-12-0950 22573885 PMC3386207

[B58] Qiu H. Wu X. Ma X. Li S. Cai Q. Ganzella M. (2024). Short-distance vesicle transport via phase separation. Cell 187 (9), 2175–2193.e2121. 10.1016/j.cell.2024.03.003 38552623

[B59] Raddum A. M. Evensen L. Hollas H. Grindheim A. K. Lorens J. B. Vedeler A. (2013). Domains I and IV of annexin A2 affect the formation and integrity of *in vitro* capillary-like networks. PLoS One 8 (3), e60281. 10.1371/journal.pone.0060281 23555942 PMC3612057

[B60] Rescher U. Ruhe D. Ludwig C. Zobiack N. Gerke V. (2004). Annexin 2 is a phosphatidylinositol (4,5)-bisphosphate binding protein recruited to actin assembly sites at cellular membranes. J. Cell Sci. 117 (16), 3473–3480. 10.1242/jcs.01208 15226372

[B61] Rescher U. Ludwig C. Konietzko V. Kharitonenkov A. Gerke V. (2008). Tyrosine phosphorylation of annexin A2 regulates Rho-mediated actin rearrangement and cell adhesion. J. Cell Sci. 121 (13), 2177–2185. 10.1242/jcs.028415 18565825

[B62] Robbins E. Gonatas N. K. (1964). The ultrastructure of a mammalian cell during the mitotic cycle. J. Cell Biol. 21 (3), 429–463. 10.1083/jcb.21.3.429 14189913 PMC2106374

[B63] Rollins K. R. Blankenship J. T. (2023). Dysregulation of the endoplasmic reticulum blocks recruitment of centrosome-associated proteins resulting in mitotic failure. Development 150 (22), dev201917. 10.1242/dev.201917 37971218 PMC10690056

[B64] Ruiz M. Bogus S. M. Ernst A. M. (2026). Biomolecular condensates in and around the ER-Golgi interface. Subcell. Biochem. 110, 221–243. 10.1007/978-3-032-06936-8_9 41240313

[B65] Sager P. R. Brown P. A. Berlin R. D. (1984). Analysis of transferrin recycling in mitotic and interphase HeLa cells by quantitative fluorescence microscopy. Cell 39 (2 Pt 1), 275–282. 10.1016/0092-8674(84)90005-9 6498936

[B66] Salina D. Bodoor K. Eckley D. M. Schroer T. A. Rattner J. B. Burke B. (2002). Cytoplasmic dynein as a facilitator of nuclear envelope breakdown. Cell 108 (1), 97–107. 10.1016/s0092-8674(01)00628-6 11792324

[B67] Sandquist J. C. Kita A. M. Bement W. M. (2011). And the dead shall rise: actin and myosin return to the spindle. Dev. Cell 21 (3), 410–419. 10.1016/j.devcel.2011.07.018 21920311 PMC3197778

[B68] Sannerud R. Marie M. Hansen B. B. Saraste J. (2008). Use of polarized PC12 cells to monitor protein localization in the early biosynthetic pathway. Methods Mol. Biol. 457, 253–265. 10.1007/978-1-59745-261-8_19 19066033

[B69] Saraste J. Marie M. (2018). Intermediate compartment (IC): from pre-Golgi vacuoles to a semi-autonomous membrane system. Histochem Cell Biol. 150 (5), 407–430. 10.1007/s00418-018-1717-2 30173361 PMC6182704

[B70] Saraste J. Prydz K. (2019). A new look at the functional organization of the Golgi ribbon. Front. Cell Dev. Biol. 7, 171. 10.3389/fcell.2019.00171 31497600 PMC6713163

[B71] Schlaitz A. L. Thompson J. Wong C. C. Yates J. R. 3rd Heald R. (2013). REEP3/4 ensure endoplasmic reticulum clearance from metaphase chromatin and proper nuclear envelope architecture. Dev. Cell 26 (3), 315–323. 10.1016/j.devcel.2013.06.016 23911198 PMC3745822

[B72] Scholey J. M. (2025). Mitotic spindle membranes. Mol. Biol. Cell 36 (4), re1. 10.1091/mbc.E24-10-0475 40067152 PMC12005112

[B73] Schweizer N. Weiss M. Maiato H. (2014). The dynamic spindle matrix. Curr. Opin. Cell Biol. 28, 1–7. 10.1016/j.ceb.2014.01.002 24491920

[B74] Seemann J. Pypaert M. Taguchi T. Malsam J. Warren G. (2002). Partitioning of the matrix fraction of the Golgi apparatus during mitosis in animal cells. Science 295 (5556), 848–851. 10.1126/science.1068064 11823640

[B75] Serio G. Margaria V. Jensen S. Oldani A. Bartek J. Bussolino F. (2011). Small GTPase Rab5 participates in chromosome congression and regulates localization of the centromere-associated protein CENP-F to kinetochores. Proc. Natl. Acad. Sci. U. S. A. 108 (42), 17337–17342. 10.1073/pnas.1103516108 21987812 PMC3198334

[B76] Shorter J. Warren G. (2002). Golgi architecture and inheritance. Annu. Rev. Cell Dev. Biol. 18, 379–420. 10.1146/annurev.cellbio.18.030602.133733 12142281

[B77] Skop A. R. Liu H. Yates J. 3rd Meyer B. J. Heald R. (2004). Dissection of the mammalian midbody proteome reveals conserved cytokinesis mechanisms. Science 305 (5680), 61–66. 10.1126/science.1097931 15166316 PMC3679889

[B78] Sridharan S. Hernandez-Armendariz A. Kurzawa N. Potel C. M. Memon D. Beltrao P. (2022). Systematic discovery of biomolecular condensate-specific protein phosphorylation. Nat. Chem. Biol. 18 (10), 1104–1114. 10.1038/s41589-022-01062-y 35864335 PMC9512703

[B79] Strand E. Hollås H. Sakya S. A. Romanyuk S. Saraste M. E. V. Grindheim A. K. (2021). Annexin A2 binds the internal ribosomal entry site of c-myc mRNA and regulates its translation. RNA Biol. 18 (Suppl. 1), 337–354. 10.1080/15476286.2021.1947648 34346292 PMC8677036

[B80] Sun M. Jia M. Ren H. Yang B. Chi W. Xin G. (2021). NuMA regulates mitotic spindle assembly, structural dynamics and function via phase separation. Nat. Commun. 12 (1), 7157. 10.1038/s41467-021-27528-6 34887424 PMC8660824

[B81] Tacheva-Grigorova S. K. Santos A. J. Boucrot E. Kirchhausen T. (2013). Clathrin-mediated endocytosis persists during unperturbed mitosis. Cell Rep. 4 (4), 659–668. 10.1016/j.celrep.2013.07.017 23954786 PMC3849811

[B82] Takahashi S. Takei T. Koga H. Takatsu H. Shin H. W. Nakayama K. (2011). Distinct roles of Rab11 and Arf6 in the regulation of Rab11-FIP3/arfophilin-1 localization in mitotic cells. Genes cells. 16 (9), 938–950. 10.1111/j.1365-2443.2011.01538.x 21790911

[B83] Takatsu H. Katoh Y. Ueda T. Waguri S. Murayama T. Takahashi S. (2013). Mitosis-coupled, microtubule-dependent clustering of endosomal vesicles around centrosomes. Cell Struct. Funct. 38 (1), 31–41. 10.1247/csf.12028 23328347

[B84] Théry M. Bornens M. (2008). Get round and stiff for mitosis. Hfsp J. 2 (2), 65–71. 10.2976/1.2895661 19404473 PMC2645575

[B85] Tiwary A. K. Zheng Y. (2019). Protein phase separation in mitosis. Curr. Opin. Cell Biol. 60, 92–98. 10.1016/j.ceb.2019.04.011 31176175 PMC6756948

[B86] Tooze J. Hollinshead M. (1992). Evidence that globular Golgi clusters in mitotic HeLa cells are clustered tubular endosomes. Eur. J. Cell Biol. 58 (2), 228–242. 1425764

[B87] Tsai M. Y. Wang S. Heidinger J. M. Shumaker D. K. Adam S. A. Goldman R. D. (2006). A mitotic lamin B matrix induced by RanGTP required for spindle assembly. Science 311 (5769), 1887–1893. 10.1126/science.1122771 16543417

[B88] Ungricht R. Kutay U. (2017). Mechanisms and functions of nuclear envelope remodelling. Nat. Rev. Mol. Cell Biol. 18 (4), 229–245. 10.1038/nrm.2016.153 28120913

[B89] Valapala M. Vishwanatha J. K. (2011). Lipid raft endocytosis and exosomal transport facilitate extracellular trafficking of annexin A2. J. Biol. Chem. 286 (35), 30911–30925. 10.1074/jbc.M111.271155 21737841 PMC3162451

[B90] Vedeler A. Hollas H. (2000). Annexin II is associated with mRNAs which may constitute a distinct subpopulation. Biochem. J. 348 (Pt 3), 565–572. 10.1042/bj3480565 10839987 PMC1221098

[B91] Vedeler A. Hollas H. Grindheim A. K. Raddum A. M. (2012). Multiple roles of annexin A2 in post-transcriptional regulation of gene expression. Curr. Prot. Pept. Sci. 13 (4), 401–412. 10.2174/138920312801619402 22708494

[B92] Vilmos P. Kristó I. Szikora S. Jankovics F. Lukácsovich T. Kari B. (2016). The actin-binding ERM protein Moesin directly regulates spindle assembly and function during mitosis. Cell Biol. Int. 40 (6), 696–707. 10.1002/cbin.10607 27006187

[B93] Wilson G. M. Fielding A. B. Simon G. C. Yu X. Andrews P. D. Hames R. S. (2005). The FIP3-Rab11 protein complex regulates recycling endosome targeting to the cleavage furrow during late cytokinesis. Mol. Biol. Cell 16 (2), 849–860. 10.1091/mbc.e04-10-0927 15601896 PMC545916

[B94] Woodruff J. B. (2018). Assembly of mitotic structures through phase separation. J. Mol. Biol. 430 (23), 4762–4772. 10.1016/j.jmb.2018.04.041 29751016

[B95] Zaal K. J. Smith C. L. Polishchuk R. S. Altan N. Cole N. B. Ellenberg J. (1999). Golgi membranes are absorbed into and reemerge from the ER during mitosis. Cell 99 (6), 589–601. 10.1016/s0092-8674(00)81548-2 10612395

[B96] Zeuschner D. Stoorvogel W. Gerke V. (2001). Association of annexin 2 with recycling endosomes requires either calcium- or cholesterol-stabilized membrane domains. Eur. J. Cell Biol. 80 (8), 499–507. 10.1078/0171-9335-00184 11561901

[B97] Zheng Y. (2010). A membranous spindle matrix orchestrates cell division. Nat. Rev. Mol. Cell Biol. 11 (7), 529–535. 10.1038/nrm2919 20520622 PMC5474289

[B98] Zobiack N. Rescher U. Ludwig C. Zeuschner D. Gerke V. (2003). The annexin 2/S100A10 complex controls the distribution of transferrin receptor-containing recycling endosomes. Mol. Biol. Cell 14 (12), 4896–4908. 10.1091/mbc.e03-06-0387 13679511 PMC284793

