# Supplementary material for: Annexin A2 and lamin B join membrane recycling compartments for the assembly of biomolecular condensates operating in mitotic partitioning
Source: Front Cell Dev Biol. 2026 Jan 15;13:1744307. doi: 10.3389/fcell.2025.1744307 (PMC12852427; doi:10.3389/fcell.2025.1744307)
Supplement: Supplementary file 3 [file DataSheet1.docx]

Grindheim et al. Supplementary Material

# 1. Supplementary Figures


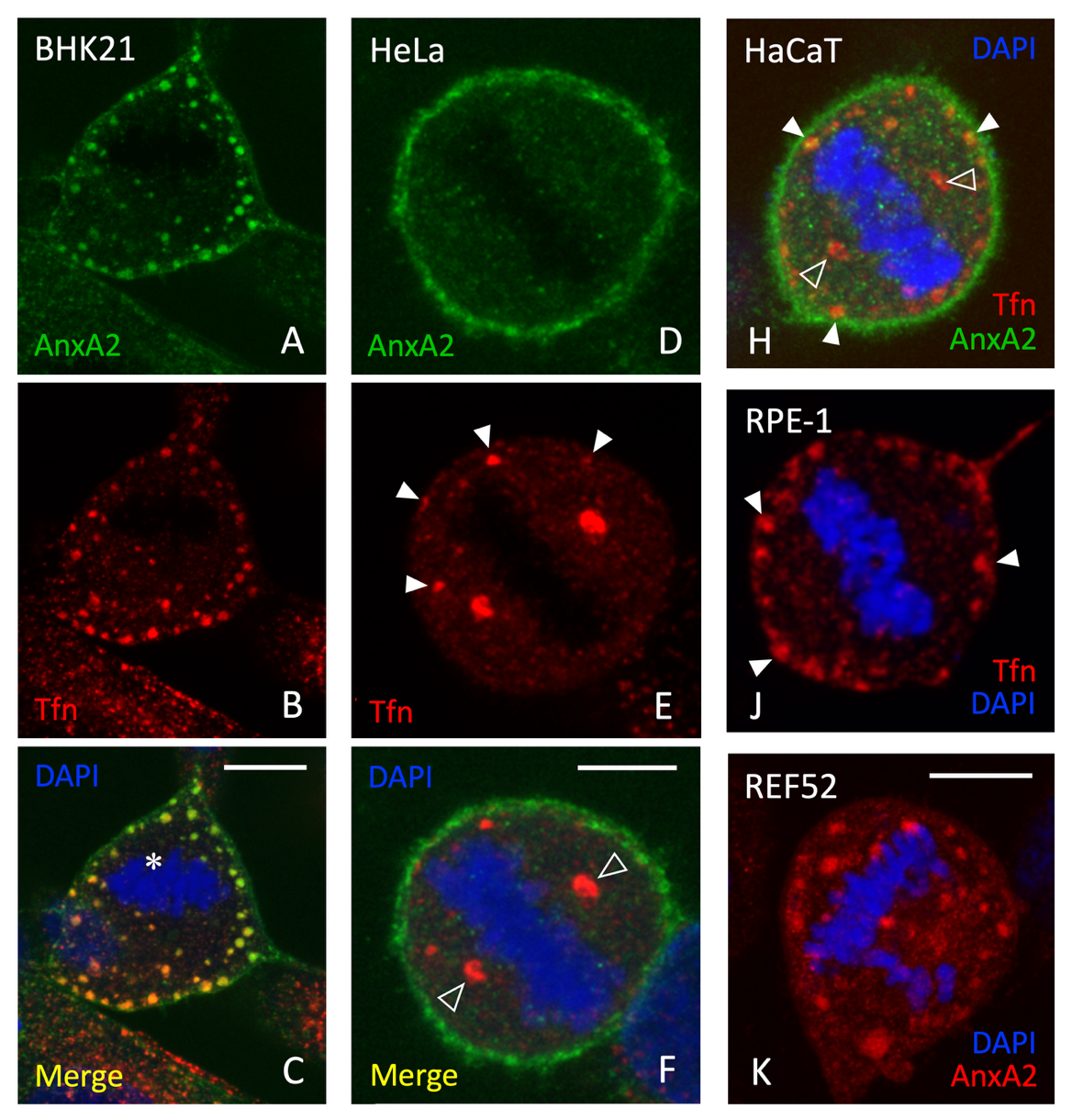


**Figure S1.** ***Demonstration of the mitotic structures in other cells.*** The presence of the structures in different cell types fixed with PLP was addressed using antibodies against AnxA2 (REF52 cells), endocytic uptake of Alexa Fluor 594-coupled Tfn (RPE-1 cells), or both (BHK21, HeLa and HaCaT cells). Note the predominantly diffuse localization of AnxA2 in the cortex of HeLa cells (D), the presence of endocytosed Tfn in only a few peripheral structures (E; white arrows) and its piling-up at the spindle poles (F; open arrowheads), as also observed in HaCaT cells (H). Scale bars: 10 µm.


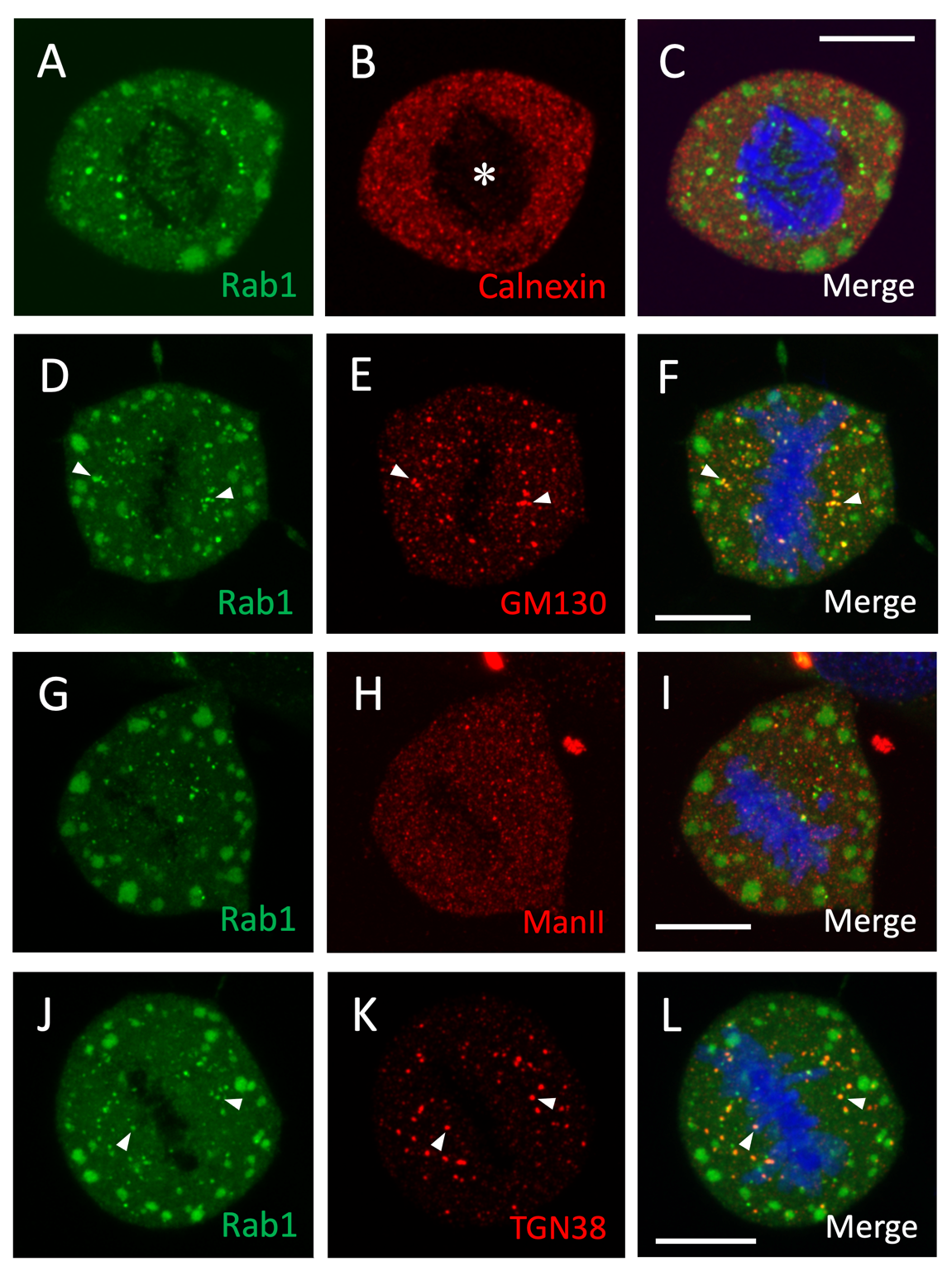


**Figure S2.** ***The mitotic structures do not contain ER or Golgi membranes*.** NRK cells expressing GFP-Rab1 were fixed with PLP and stained with antibodies against calnexin (ER; panels A-C), GM130 (*cis*-Golgi; panels D-F), mannosidase II (*medial*-Golgi; panels G-I) or TGN38 (*trans-*Golgi/TGN; panels J-L). Note the absence of these secretory organelle markers from the Rab1-positive peripheral structures of metaphase cells. Calnexin is absent from spindle region (panel B, asterisk), while the diffuse staining for mannosidase II corresponds to the vesicular “Golgi haze”. The Rab1 effector GM130, and interestingly also TGN38, partially colocalize with the Rab1-positive IC elements at the spindle poles (white arrowheads). Scale bars: 10 µm.


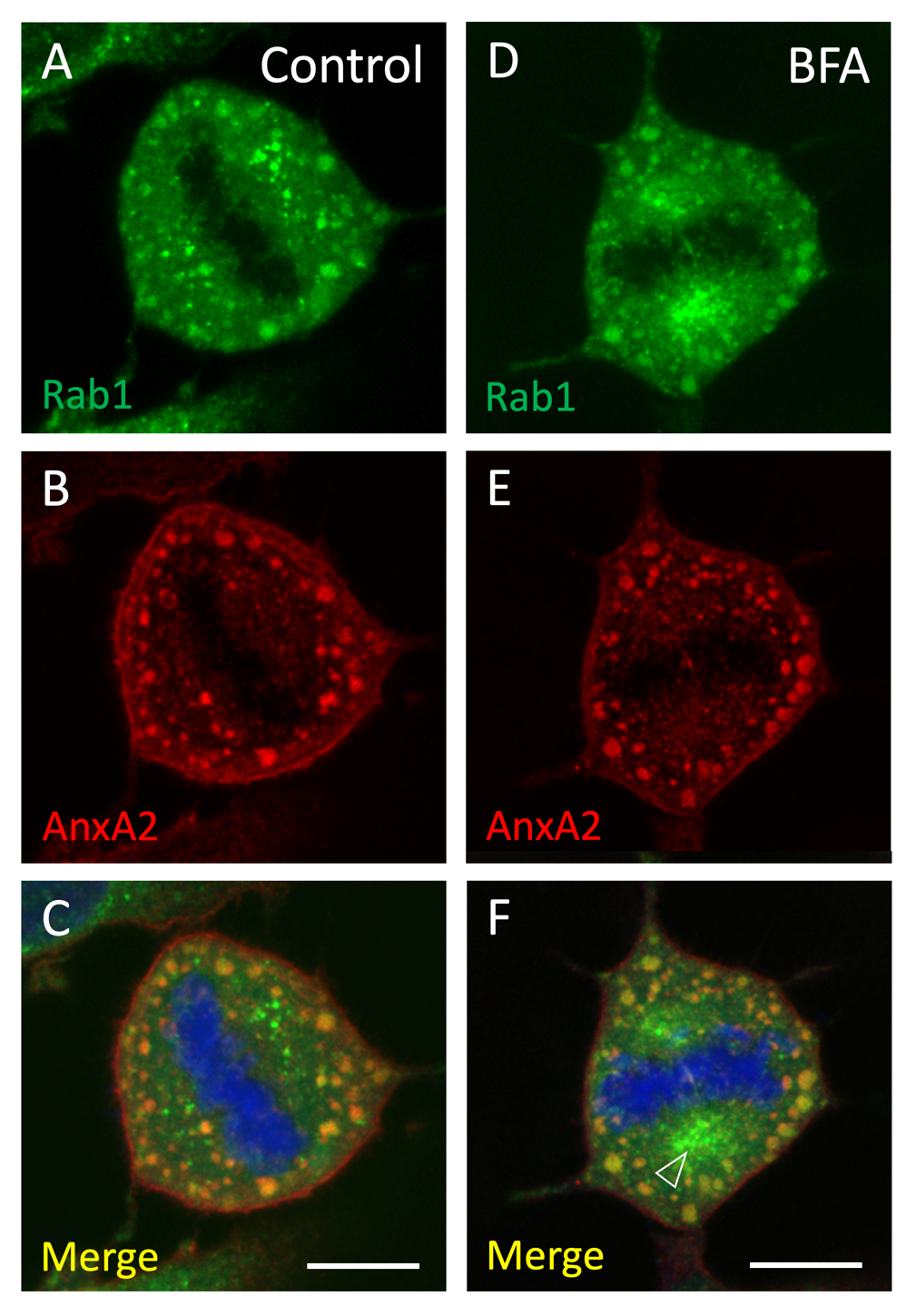


**Supplementary Figure 3.** *The mitotic structures are not affected by brefeldin A (BFA).* Control NRK cells expressing GFP-Rab1 (panels A-C), or cells treated for 30 min with BFA (5 µg/ml) (panels D-F) were fixed with PLP and stained for AnxA2 (red). BFA does not appear to influence the structure or localization of the large peripheral structures, or appearance of the condensed chromosomes in metaphase cells, but by releasing membrane-bound COPI coats results in the tubulation of the IC elements at the spindle poles (open arrow). Scale bars: 10 µm.


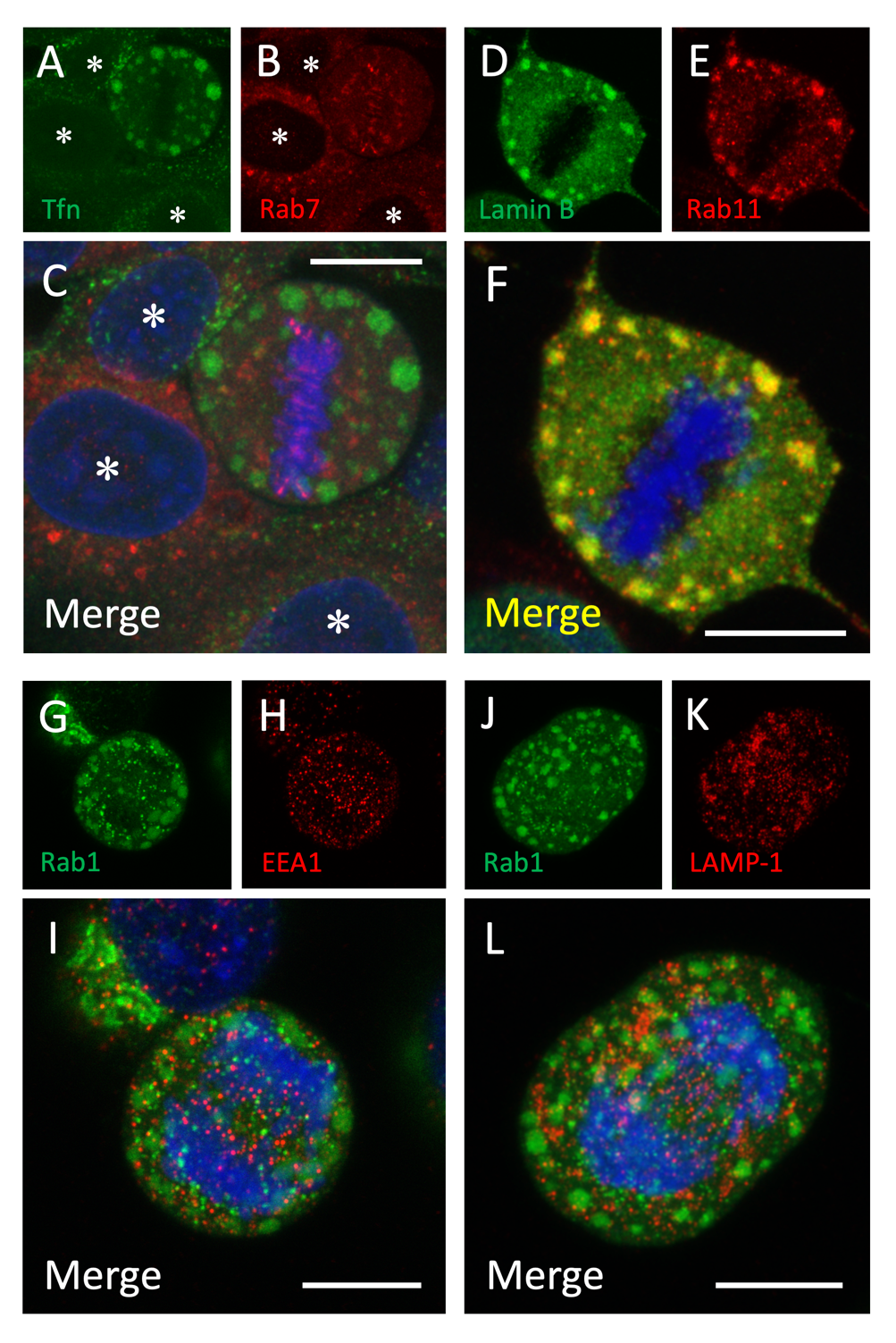


**Supplementary Figure 4.** *The mitotic structures contain Rab11-positive REs but lack early or late endosomes or lysosomes.* The localizations of different endosomal markers, including Rab7 (panels A-C), Rab11 (panels D-F), as well as EEA1 and LAMP-1 (panels G*-*I and J-L) in metaphase cells were compared with markers of the large peripheral structures – transferrin (Tfn), lamin B and Rab1, respectively. In contrast to the RE marker Rab11, the early (EEA1) or late endosomal/lysosomal markers (Rab7/LAMP-1) do not localize to the large mitotic structures. Interphase cells are marked with asterisks. Scale bars: 10 µm.

##
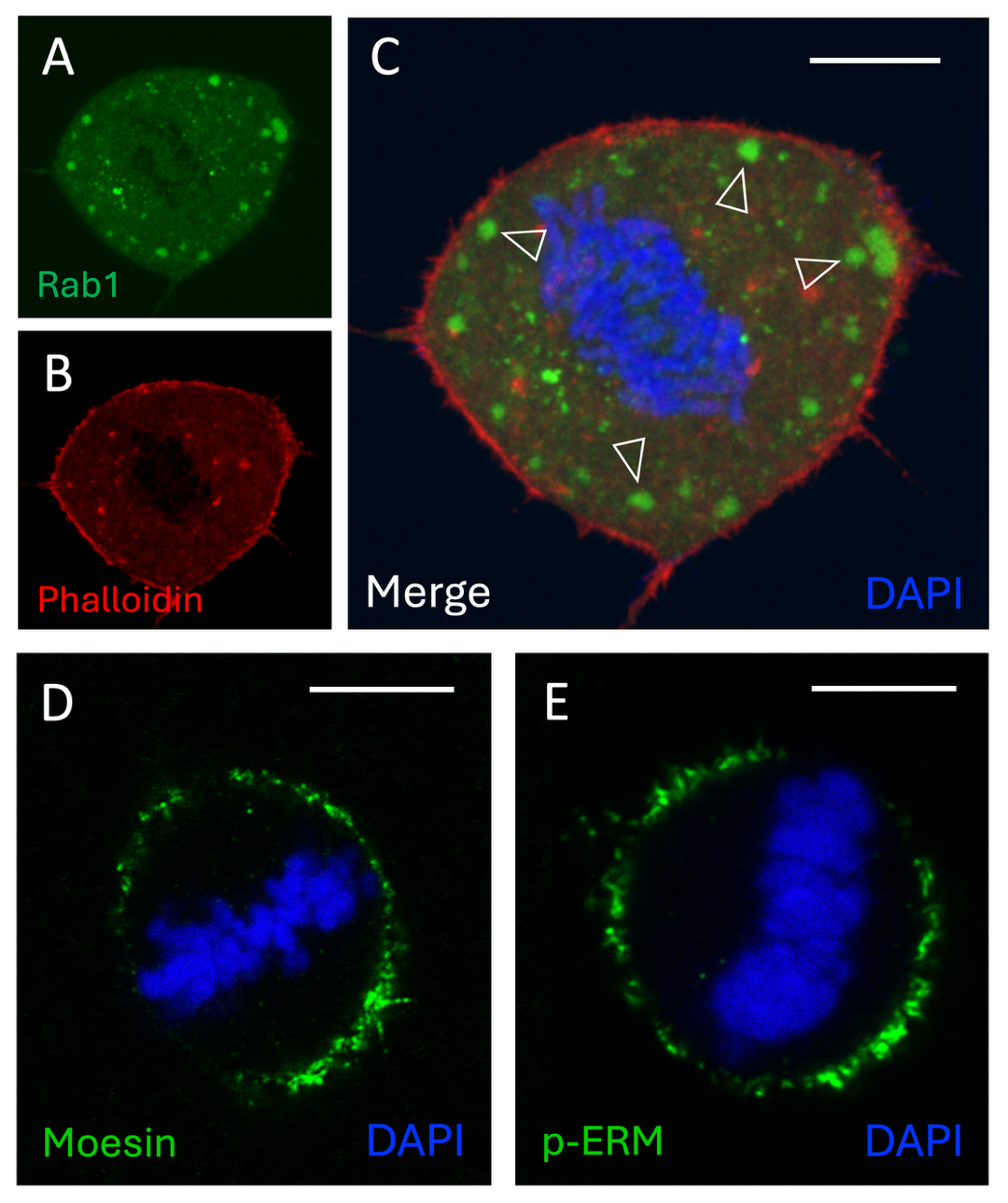


**Figure S5. *The peripheral mitotic structures do not contain actin filaments or ERM proteins*.** The GFP-Rab1 expressing NRK cells or parental NRK cells were stained with Alexa Fluor 594-coupled phalloidin or antibodies against moesin or phosphorylated ERM proteins, respectively. DNA staining was with DAPI. The peripheral mitotic structures in panel C are marked with open arrowheads. Scale bars: 10 µm.


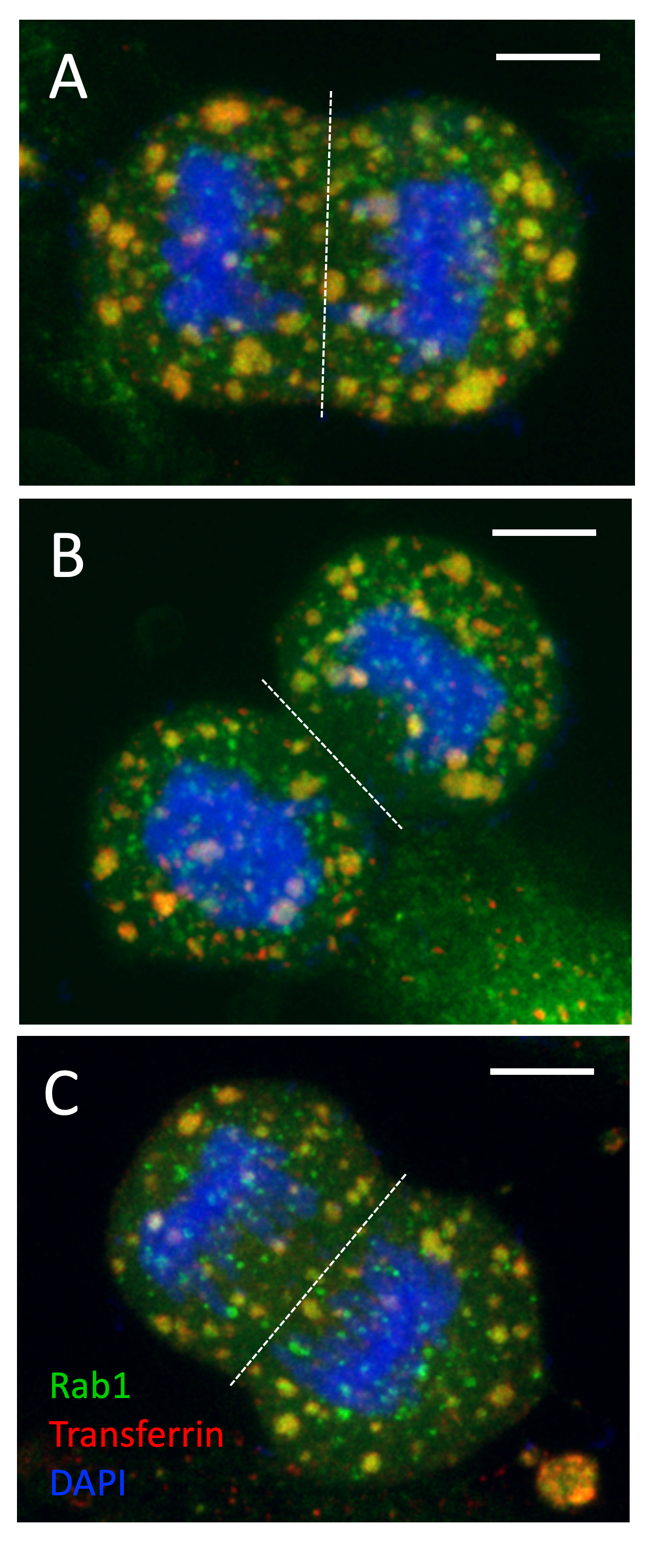


**Figure S6. *Mitotic partitioning of the condensates.*** NRK cells expressing GFP-Rab1 were subjected to endocytic uptake of Alexa Fluor 594-coupled transferrin and stained with DAPI*.* In the late anaphase cells shown, the predominantly peripheral large mitotic condensates – double-stained for Rab1 and Tfn (yellow) – appear to roughly equally segregate between the daughter cells, separated by the forming cleavage furrow (dashed lines). Scale bars: 10 µm.

## Legends for Supplementary Movies

**Supplementary Movie 1.** *Animation demonstrating the peripheral localization of the mitotic condensates in a metaphase cell*. NRK cells expressing GRP-Rab1 were stained with antibodies against AnxA2 and with DAPI. The Rab1- and AnxA2-positive mitotic structures (yellow) and condensed chromosomes (blue) were surface-rendered, and the movie was prepared using the Imaris software. Note also the localization of the Rab1-containing IC elements (green) at the spindle poles.

**Supplementary Movie 2.** *Animation showing mitotic partitioning of the condensates.* NRK cells at late anaphase, expressing GRP-Rab1 were stained with antibodies against AnxA2 and with DAPI. The condensed chromosomes (blue) and the Rab1- and AnxA2-positive large mitotic structures in the forming daughter cells were surface-rendered, and the latter were differentially pseudo-coloured (yellow or blue). The movie was prepared using the Imaris software.
